# Sequence analysis of the cDNA encoding for SpCTx: a lethal factor from scorpionfish venom (*Scorpaena plumieri*)

**DOI:** 10.1186/s40409-018-0158-7

**Published:** 2018-08-29

**Authors:** Fábio L. S. Costa, Maria Elena De Lima, Suely G. Figueiredo, Rafaela S. Ferreira, Núbia S. Prates, Tetsu Sakamoto, Carlos E. Salas

**Affiliations:** 1 Departamento de Bioquímica e Imunologia, Instituto de Ciências Biológicas, Universidade Federal de Minas Gerais, Av. Antônio Carlos, 6627, Pampulha, Belo Horizonte, MG 31270-901 Brazil; 2 Departamento de Ciências Fisiológicas, Universidade Federal do Espírito Santo, Vitória, ES Brazil

**Keywords:** cDNA, Lethal factor, *Scorpaena plumieri*, Scorpionfish, Venom gland

## Abstract

**Background:**

Lethal factors are multifunctional oligomeric proteins found in the venomous apparatus of Scorpaeniformes fish. These toxins elicit not only an array of biological responses in vitro but also cardiovascular disorders and strong hemolytic, nociceptive and edematogenic activities in vivo. This work describes the cloning and molecular identification of two toxin subunits, denominated Sp-CTx-α and Sp-CTx-β, from scorpionfish venom (*Scorpaena plumieri*).

**Methods:**

The primary structures were deduced after cDNA amplification by PCR with primers from conserved sequences described in Scorpaeniformes toxins. Following DNA sequencing and bioinformatic analysis, the tridimensional structures of both subunits were modeled.

**Results:**

The translated sequences (702 amino acids, each subunit) show homology with other lethal factors, while alignment between Sp-CTx-α and Sp-CTx-β shows 54% identity. The subunits lack N-terminal signal sequences and display masses of approximately 80 kDa each. Both Sp-CTx subunits display a B30.2/SPRY domain at the C-terminal region with typically conserved motifs as described in these toxins. Secondary structure prediction identified six α-helices 18 residues long in both α and β subunits, some of them amphiphilic with their N-terminal flanked by many basic residues, creating a cationic site associated with the cytolytic activity of these toxins. Antimicrobial potential sites were identified in Sp-CTx and share some features with other peptides presenting variable and broad-spectrum activity. A phylogenetic tree built to represent these toxins supports the proximity between scorpionfish, lionfish and stonefish.

**Conclusion:**

The study identified a putative toxin protein whose primary structure is similar to other fish toxins and with potential for production of antivenom against scorpionfish envenomation in Brazil. As a prelude to structure-function studies, we propose that the toxin is structurally related to pore-forming marine toxins.

**Electronic supplementary material:**

The online version of this article (10.1186/s40409-018-0158-7) contains supplementary material, which is available to authorized users.

## Background

Scorpaeniformes from the families Scorpaenidae and Synanceiidae are the most venomous marine fishes known to date. Their venom apparatus encompasses dorsal, anal and pelvic fin spines associated with venom-containing tissues glands [1]. Occasional envenomation occurs by accidental poisoning by fish spines. Clinical and pharmacological studies suggest that active components of fish venom exhibit cytolytic (hemolytic), inflammatory, neuromuscular and pronounced cardiovascular activities [2–5].

Scorpionfish members of the genus *Scorpaena* inhabit shallow waters of the tropical Atlantic Coast. *Scorpaena plumieri*, known in Brazil as *“aniquim”*, *“mamangá”* or *“moréia-atí”*, exhibits disguising coloration that predisposes humans to poisoning along the Brazilian shore [6]. An array of symptoms including excruciating pain at the site of the puncture, edema and cardiovascular disorders are observed following envenoming [7].

Many of the symptoms associated with injury caused by Scorpaeniformes are attributable to multifunctional proteins, described as “lethal factors” identified in the venom. Due to their strong hemolytic activity, these proteins have been designated as cytolytic toxins or “multifunctional cytolysins” (for a review, see [4, 8]). It was demonstrated that the hemolytic effect of these toxins is due to pore formation on the cell membrane of erythrocytes [9–12].

So far, cytolysins have been identified in the following groups: *Pterois* [13, 14], *Scorpaenopsis*, *Sebastiscus* and *Sebastapistes* [15] and *Scorpaena* [16] from the Scorpaenidae family, *Hypodytes* from the Tetraogidae family, *Siganus fuscescens* from the Siganidae family [17] and *Inimicus* [14] and *Synanceia* [18–20] from the family Synanceiidae.

The toxins are 148–160 kDa proteins composed by two homologous subunits, designated as α and β, that remain associated via non-covalent interaction creating a dimeric structure. The domains MACPF/CDC (Membrane Attack Complex-Perforin/Cholesterol-Dependent Cytolysin), known for forming large, ring-shaped supramolecular oligomeric pore complexes on erythrocyte membranes, represent an ancient pore-forming superfamily [10, 19, 20].

The cytolytic toxin (Sp-CTx) was purified from venom of the scorpionfish *S. plumieri* [11, 16]. It displays vasorelaxant activity and induces disorders in the cardiovascular system by an increase in sarcolemmal Ca^+ 2^, partially caused by release of endogenous noradrenaline [21, 22]. Sp-CTx is a dimeric glycoprotein (≈ 75 kDa/subunit); its tryptic digestion yields peptide fragments whose Open Reading Frame (ORF) confirms its similarity to fish cytolysins [11, 16].

A striking property shared by fish venoms is their ability to induce hemolysis in vitro, arguing for a functional resemblance. The structural similarity between fish venoms was evident as most toxins were disabled upon reaction with horse-derived stonefish antivenom (SFAV) raised against crude venom of the stonefish *Synanceia verrucosa* (Commonwealth Serum Laboratories, Melbourne, Australia) [13, 14, 19, 20, 23–26]. The immune cross-reactivity among Scorpaeniformes toxins suggests that they share a common evolutionary ancestor. Based on these similarities, the design of DNA primers derived from the structure of stonefish toxin was instrumental for inferring the structure of *S. verrucosa* toxin [19, 27]. A similar strategy was applied to determine the primary structures of toxins from lionfish, waspfish and rabbitfish [14, 17], barchin scorpionfish, tassled scorpionfish and false kelpfish [15].

We previously described the production and partial characterization of a cDNA library from venomous tissue of *S. plumieri*, by using the random sequencing approach, and generated hundreds of partial sequences [28]. This study aims to identify the coding sequences for *S. plumieri* toxin, and to verify the presence of determinants attributable to the protein that could be responsible for the pharmacological effects of this toxin. To find the mRNA encoding for the lethal factor in *S. plumieri*, we have used the library or the cDNAs source of this library and primers from conserved regions of the toxin to produce the in silico full amino-acid sequence of α- and β-subunits of Sp-CTx. We further analyzed structural features of the hypothetic protein and the similarities with other fish venom toxins.

## Methods

### Biological specimens

Three live specimens of the scorpionfish *S. plumieri* (15–30 cm, length) were collected by a local fisherman off the Coast at Espírito Santo, Brazil and kept in an aquarium for a short duration prior to dissection. Fishing was authorized by the Instituto Brasileiro do Meio Ambiente e dos Recursos Naturais Renováveis – IBAMA (the Brazilian Public Agency for Environment Affairs). Glands tissue was dissected from the dorsal, pelvic and caudal ray-fin structures and kept in liquid N_2_ during homogenization in a grinder mill.

### cDNA library construction

Total RNA was obtained from excised venom glands using the guanidinium isothiocyanate extraction procedure described by [29]. Poly(A)^+^ RNA was isolated by oligo(dT)-cellulose chromatography (mRNA Isolation Kit, Agilent Technologies, Inc. USA). Five μg of RNA were transcribed into cDNA using the ZAP cDNA synthesis kit (ZAP-cDNA Gigapack III gold cloning kit, GE, USA).

### RT-PCR procedure

A polymerase chain reaction was performed to amplify DNA from the excised bacteriophage library (~ 10^6^ pfus) or from the cDNA synthesized from 5 μg of total RNA or 500 ng of mRNA chromatographically purified following cDNA synthesis (GE Healthcare Life Sciences, USA), according to the manufacturer’s instructions.

DNA amplification was performed using Platinum® *Taq* DNA Polymerase (Invitrogen™, Life Technologies, Inc. USA) under the following conditions: pre-incubation at 94 °C for 5 min; 35 cycles consisting of denaturation at 94 °C for 30 s, annealing at 45–65 °C (*Tm* depending of the primer) for 30 s; extension at 72 °C for 1–2 min and final extension at 72 °C for 5 min. Amplified products were subcloned into pCR®8/GW/TOPO® TA Cloning with One Shot® TOP10 *E. coli* kit (Invitrogen™, Life Technologies, Inc.). The DNA of plasmid clones was isolated as described by Sambrook & Russell [30] and used for sequencing. Each PCR fragment encoding a putative region of the toxin was cloned and the consensus sequence of at least 3–4 replicates assembled into the final sequence.

### Primer design

**Fig. 1:**
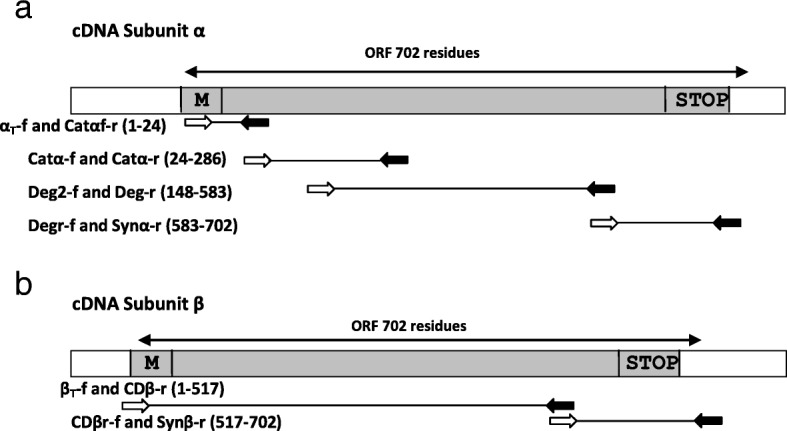
Schematic cloning representation of α- and β-subunits of Sp-CTx. Forward and reverse primers are indicated by white and black arrows, respectively. The sequence of primers is shown in Table 1. Amino-acid positions are relative to the primary structures of cDNAs from Synanceia. The positions of the arrows indicate the approximate size of the putative fragment. Initiation codon (M) and stop codon (STOP). **a**: Union of putative fragments in α-subunit was obtained by PCRs (αT-f and Catαf-r, Catα-f and Catα-r, Deg2-f and Deg-r, Degr-f and Synα-r). **b**: Union of putative fragments in β-subunit (βT-f and CDβ-r were isolated from the cDNA library and CDβr-f and Synβ-r obtained by PCR)

Initially, primers were designed based on conserved sequences from toxins already described in other Scorpaeniform species. The nucleotide sequences of primers used in experiments are summarized in Table 1. A total of twelve primers (eight for α-subunit and four for β-subunit) were employed to fully characterize the DNA encoding both subunits (Fig. 1). DNA primers Deg (forward and reverse) were used as described by Kiriake & Shiomi [13] to identify lionfish toxins while remaining primers were based on toxin sequences from stonefish [5, 9, 19, 20].

**Table 1:** Nucleotide sequences of primers used for RT-PCR and cloning experiments

Primer identification	Nucleotide sequence of primer	Nucleotide position^a^
Subunit α		
α_T_-f	5’-ATGTCTTCAGATTTGGTAATGCCT-3’	60–83
Catα-f	5’-CGCAGAGAGAAACTGATCCCA-3’	126–146
Catαf-r	5’-TGGGATCAGTTTCTCTCTGCG-3’	126–146
Deg2-f	5’-GGGGCMAATGCYTTCTTTGT-3’	498–517
Catα-r	5’-ATTGGCTCTCCTCTTCAGTTT-3’	900–921
Degr-f	5’-ATTACTGGGAGGTGGAGTGG-3’	1790-1809
Deg-r	5’-CCACTCYAMCTCCCAGTAAT-3’	1790-1809
Synα-r	5’-TYAAAGTAATCTGASAGTTCC-3’	2151-2171
Subunit β		
β_Total_-f	5’-GTTGGAGTCATGCCTTCAGAC-3’	52–71
CDβ-r	5’-GAGCTCACAGTCATACCA-3’	1621-1638
CDβr-f	5’-TGGTATGACTGTGAGCTC-3’	1621-1638
Synβ-r	5’-TAGTTTAATTTGACCATT-3’	2139-2157

^a^Numbering is based on nucleotide sequence from stonustoxin (*Synanceia horrida*), accession numbers GenBank: U36237 and U32516 [18]

Designations of the primers were based on reported DNA sequences corresponding to regions 60–83 (α_T_-f), 126–146 (Catα-f or Catαf-r), 498–517 (Deg2-f), 1790–1809 (Deg-r or Degr-f) and (2151-2171) Synα-r from α-subunit and (52–71) β_T_-f, 1621–1638 (CDβ-r or CDβr-f), and 2139–2157 (Synβ-r) from β-subunit (Fig. 1).

### Comparative modeling

Comparative models of the Sp-CTx (α- and β-subunits) were constructed using the Automated Mode of SWISS-MODEL server [31]. The target sequences were used for identification of templates based on Blast and HHblits. The crystal structures of stonustoxin subunits α (PDB ID:4WVM_A) and β (PDB ID:4WVM_B), at 3.1 Å resolution, were used for modeling of Sp-CTx subunits. The alignment between target and template sequences was conducted to generate 3D models. The stereochemical quality of the models was determined by Ramachandran plot assessment generated by RAMPAGE [32]. The models were further evaluated through ProSA [33] and QMEAN statistical parameters [34]. We also calculated the RMSD values between the models and their corresponding template.

The HADDOCK 2.2 web server [35] was used for protein-protein docking of modeled structures. During the docking procedure, HADDOCK incorporated information about interacting residues at the interface of the protein complex. Therefore, before docking, contacts were identified with the InterProSurf web server [36], using template structures as an input to predict interacting residues.

### Sequence and analysis of clones

Colonies grown overnight in ampicillin-supplemented medium at 37 °C were randomly selected. Plasmid DNA was isolated by the alkaline lysis method [30].

DNA sequences were obtained in the automated sequencer 3.100 Genetic Analyzer System using BigDye™ Terminator v1.1, v3.1 Ready Reaction Mix (Applied Biosystems Inc., Foster City, CA, USA) in the presence of M13 forward primer or its reverse. Analysis of data was carried out using the software Phred for base calling and the quality score cutoff was set at 10 [37]. The nucleotide sequences from the vector, adaptors and *Escherichia coli* DNA were removed by the program VecScreen (http://www.ncbi.nlm.nih.gov/tools/vecscreen).

Amino-acid sequences of toxin transcripts were deduced via the program Open Reading Frame (ORF) Finder (https://www.ncbi.nlm.nih.gov/orffinder/). The isoelectric point (pI) and molecular mass (MM) from derived sequences were computed by the software Swiss-Prot/TrEMBL located in Expasy.

The amphiphilicity, α-helices, glycosylation sites and peptide signal sequences in Sp-CTx were analyzed by the programs PSIPRED Protein Sequence Analysis Workbench (UCL Department of Computer Science), NETNGLYC (http://www.cbs.dtu.dk/services/NetNGlyc) and SignalP 4.0 [38], respectively. Cytolytic sites in α-helices were predicted by designing a Helical Wheel as described by Schiffer & Edmundson [39] and using the program (http://lbqp.unb.br/NetWheels) [40].

### Phylogenetic analysis

Putative orthologues of Sp-CTx were identified by submitting derived protein sequences as queries to the BLASTP algorithm [41] on the NCBI webserver (https://blast.ncbi.nlm.nih.gov/Blast.cgi) employing the non-redundant protein sequences (nr) database. From BLASTP retrieved protein accessions, we selected those accessions displaying a high similarity score with at least one of the query sequences (coverage > 80%; identity > 50%) and pertaining to one of the species known to be venomous. Sequences were submitted to MUSCLE [42] and then to the Neighbor-Joining algorithm (bootstrap replicates: 500; substitution model: Maximum Composite Likelihood), both implemented in MEGA7 [43], for sequence alignment and phylogenetic tree creation, respectively. For tree rooting analysis, we included the Stonustoxin subunit β-like protein from *Clupea harengus* (accession number: XP_012674574.1) and considered it an outgroup.

## Results

### Cloning and sequencing of cDNAs encoding α- and β-subunits of Sp-CTx

Initially, we designed the set of primers (Catα_f-r_) coding for the region containing many cationic residues apparently involved in the hemolytic activity in Scorpaeniformes [44]. Using Catα primers (Fig. 1a) and cDNA *S. plumieri* as the template, a PCR fragment of approximately 800 bp was amplified and cloned into pCR8/GW/TOPO. The sequenced fragment contained an ORF encoding 265 amino-acid residues that aligned between positions 24–286 with α-subunits in Scorpaeniform toxins found at the NCBI databank.

To characterize the N-terminal region, a reverse complement of Catα primer was designed and combined with α_T_-f primer to produce an amplicon of 100 bp. After cloning and sequencing, this fragment generated an ORF of 24 residues corresponding to the N-terminal of the Sp-CTx α-subunit.

The C-terminal of Sp-CTx-α was identified when combining the complement of a Degr primer with Synα-r primer to yield a 400 bp fragment (Fig. 1a). After cloning and sequencing, a 126-amino-acid fragment was identified and aligned to positions 583–584 of subunit-α from fish toxins. In this fragment we identified three termination codons (TAA) in frame, at the end of the sequence.

The identification of Sp-CTx-β followed PCR of the excised library with primers β_T_-f and CDβ-r (Fig. 1b). After subcloning and sequencing, a 1545 bp PCR product yielded an ORF encoding a 515-amino-acid polypeptide sharing 81% identity with β-subunit of *Pterois*. To determine the C-terminal portion of Sp-CTx-β, a complement of CDβr-f primer was designed and combined with Synβ-r primer in PCRs using a cDNA template from *S. plumieri* (Fig. 1b). The resulting 600 bp fragment was cloned; and its sequence identified an ORF of 555 bp corresponding to 185 amino-acid residues located at C-terminals in β-subunits.

Several primers were designed to attempt identification of the internal regions of Sp-CTx-α and β under different PCR conditions (data not shown); one of them (Deg2-f, Deg-r) produced an amplicon of 1500 bp that was cloned and sequenced. Two related sequences were identified that aligned with internal regions of Sp-CTx-α (1,365 bp - 455 residues) and Sp-CTx-β (1,104 bp - 368 residues). Assemblage of overlapping fragments produced the entire sequence from Sp-CTx-α and Sp-CTx-β as expected for Scorpaeniformes toxins.

### Nucleotide sequence of α- and β-subunits of Sp-CTx

**Fig. 2:**
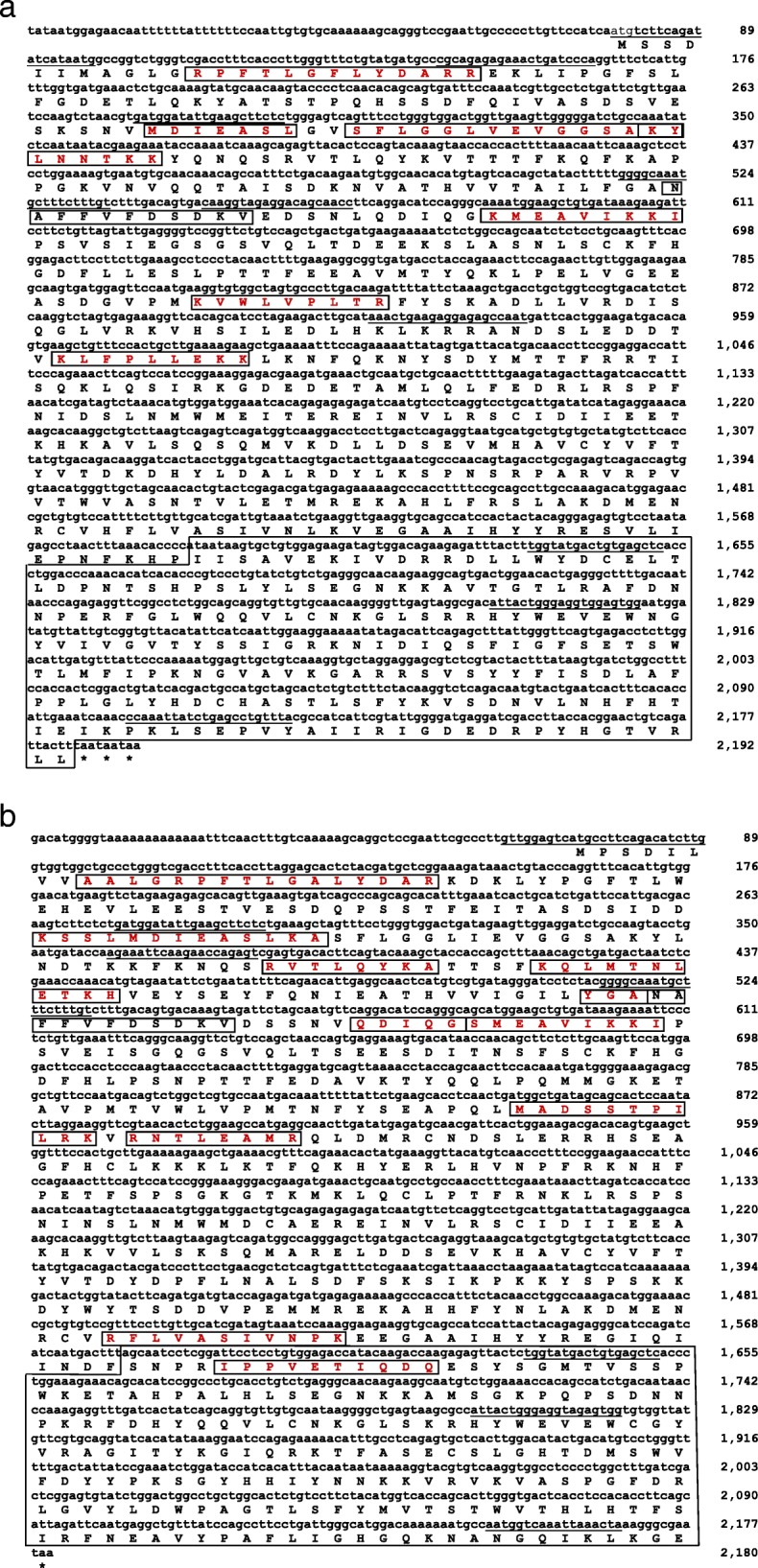
Nucleotide and deduced amino-acid sequences of cDNAs encoding Sp-CTx-α **a** and β-subunit **b**. Single-letter amino-acid notation is used. Underlined sequences refer to primers; boxed sequences were identical to peptide sequences isolated from tryptic digestion of purified Sp-CTx toxin [11]. Stop codons in frame are indicated by asterisks. The B30.2/SPRY domain is boxed. The nucleotide sequences for α- and β-subunits from *S. plumieri* have been deposited in the DDBJ/EMBL/GenBank nucleotide sequence databases under accession numbers 2,052,576 MG053103 and MG53104, respectively

Figure 2a shows the assembled Sp-CTx-α sequence containing 2192 bp. The 5′-untranslated region of this sequence contains the initiation codon located at position 78, followed by an ORF encompassing 2106 bp encoding 702 amino-acid residues in frame with three stop codons in tandem, comprising the beginning of the poly A tail at the 3′-untranslated region. In this sequence the initial ATG (Met) is followed by two Ser, and the last two amino acids before the stop codons (TAA) are Leu.

In Sp-CTx-β, the initial ATG codon was found in position 72, followed by an ORF containing 2106 bp (Fig. 2b). The initial coding ATG is followed by Pro and Ser; the 3′-terminal contains GGC-GAA (Gly-Glu) before the single stop codon (TAA). However, the poly A tail was not identified in the 3′-untranslated region. No signal peptides were identified in the N-terminal regions of Sp-CTx-α or Sp-CTx-β.

The sequences of Sp-CTx subunits were deposited in the EMBL Nucleotide Sequence Database (DDBJ/EMBL/GenBank nucleotide sequence databases) under the following accession numbers: Seq1 MG053103/AVI44916 for the α-subunit and Seq2 MG53104/AVI44917 for the β-subunit of *S. plumieri*.

### Amino-acid sequence of α- and β-subunits of Sp-CTx

**Fig. 3:**
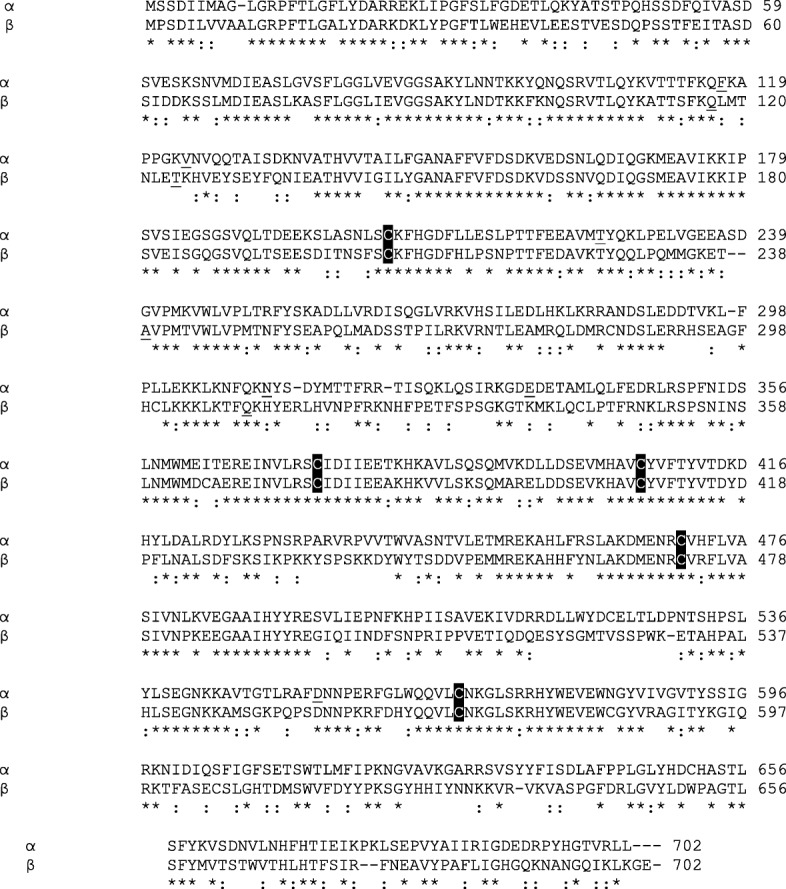
Sequence alignment between Sp-CTx-α and β-subunits. Sequences were aligned using the ClustalW2 EBI. Single-letter amino-acid notation is used. Amino acids are numbered beginning at the assumed N-terminal Met for both subunits. Identical residues are shown by asterisks, whereas conservative substitutions are indicated by colons. Conserved cysteine residues are highlighted in white on a black background. An amino acid with potential for glycosylation is underlined

A comparison between deduced amino-acid sequences of Sp-CTxs α and β evidenced 54% identity confirming their relatedness. Several insertions/deletions of one or two amino acids at various positions are detected in both subunits. Sp-CTx-α contain 7 cysteinyl residues while 11 cysteinyl are found in Sp-CTx-β, five of which (in positions 204, 374, 406, 470 and 568) are preserved in both subunits (Fig. 3). The deduced subunit-α has a theoretical molecular mass of 79,801 kDa with pI 6.70, while subunit-β has 80,126 kDa and pI 7.88.

Additional file 1 shows the deduced amino-acid sequences of Sp-CTxs and their alignment with toxins from three species of scorpionfish (*Sebastapistes strongia*, *Scorpaenopsis oxycephala* and *Sebastiscus marmoratus*), three species of lionfish (*Pterois lunulata*, *Pterois volitans* and *Pterois antennata*), two species of stonefish (*Synanceia verrucosa* and *Syanceia horrida*), one species of waspfish (*Hypodytes rubripinnis*) and one species of devil stinger (*Inimicus japonicus*). The alignment shows that 176 residues (24.5%), out of 717 amino acids (including gaps) are conserved in all toxins.

The amino-acid identities among these toxins are summarized in Table 2. It is shown that the identities between β subunits are somewhat stronger than for α-subunits. A strong identity was observed between the α-subunit in *P. lunulata* (99%) and the α-subunits from *P. volitans* and *P. antennata*. Overall, the *S. plumieri* toxin identities are stronger with those of scorpionfishes (*Scorpaenopsis oxycephala*, *Sebastapistes strongia* and *Sebastiscus marmoratus*), lionfishes (*P. lunulata*, *P. volitans* and *P. antennata*) followed by waspfish (*H. rubripinnis*), stonefish (*S. verrucosa* and *S. horrida*) and devil stinger (*I. japonicus*) toxins. The identities between Sp-CTx-α or -β subunits and the corresponding counterparts listed in Table 2 show that Sp-CTx-β shares 84% identity with toxin-β in scorpionfish *S. oxicephala* and 83% with *S. strongia*; meanwhile, the identity of Sp-CTx-α is 67% with α-subunit from *S. oxicephala* and 66% with α-subunit from *S. strongia*. The identity between subunits from the same species ranks around 47–54%; the latter corresponds to the identity between subunits α and β in *S. plumieri*. Meanwhile, within the *Pterois* group the identities between α and β subunits attain 80–82%.

**Table 2:** A comparison of the amino-acid sequence identities between Sp-CTx α-β and other fish toxins

Toxin		Amino-acid sequence identity (%)																							
		Scorpaeniformes																						Perciformes	
		*S. plumieri*		*S. marmoratus*		*S. oxycephala*		*S. strongia*		*P. lunulata*		*P. volitans*		*P. antennata*		*H. rubripinnis*		*S. horrida*		*S. verrucosa*		*I. japonicus*		*S. fuscescens*	
		α	β	α	β	α	β	α	β	α	β	α	β	α	β	α	β	α	β	α	β	α	β	α	β
*S. plumieri*	α	100																							
	β	54	100																						
*S. marmoratus*	α	58	46	100																					
	β	60	75	51	100																				
*S. oxycephala*	α	67	45	77	50	100																			
	β	62	84	52	81	50	100																		
*S. strongia*	α	66	44	75	49	92	49	100																	
	β	62	83	51	82	50	94	49	100																
*P. lunulata*	α	58	68	50	75	48	73	48	74	100															
	β	58	74	50	81	49	79	48	80	82	100														
*P. volitans*	α	58	68	50	75	48	73	48	74	99	82	100													
	β	58	73	50	80	49	79	48	79	81	98	81	100												
*P. antennata*	α	58	67	50	75	48	72	47	74	99	82	99	81	100											
	β	58	74	51	81	49	79	48	80	80	98	81	99	80	100										
*H. rubripinnis*	α	55	45	71	49	67	50	65	48	46	47	46	47	46	48	100									
	β	55	69	50	76	48	73	47	73	72	77	72	77	72	77	47	100								
*S. horrida*	α	55	45	71	48	65	50	64	48	47	48	47	48	47	48	83	48	100							
	β	54	68	49	74	49	73	47	72	69	73	69	73	68	73	47	83	48	100						
*S. verrucosa*	α	55	45	73	51	67	50	66	49	48	50	48	50	48	50	83	48	87	49	100					
	β	54	68	50	74	49	73	47	72	69	73	69	73	69	74	47	84	49	95	50	100				
*I. japonicus*	α	53	45	71	49	66	49	65	48	48	68	48	68	48	68	81	47	84	49	90	50	100			
	β	53	68	48	74	48	72	47	72	49	73	50	73	50	73	48	83	47	90	48	90	48	100		
*S. fuscescens*	α	51	62	48	66	47	64	47	65	63	66	63	65	62	66	45	65	46	65	46	65	45	64	100	
	β	41	42	44	44	43	44	43	44	42	43	42	42	41	43	40	41	41	42	42	42	42	42	43	100

The PROSITE tool [45] revealed the presence of a B30.2/SPRY domain containing 197–198 residues at the C-terminal region on each subunit, although the amino-acid sequences within these domains are somewhat variable.

### Predicted cytolytic domains

The cytolytic activity of many proteins is frequently related to the presence of amphiphilic α-helices displaying cationic sites (basic residues) flanked by hydrophobic surfaces that induce monomer aggregates able to form pores [44, 46].

**Fig. 4:**
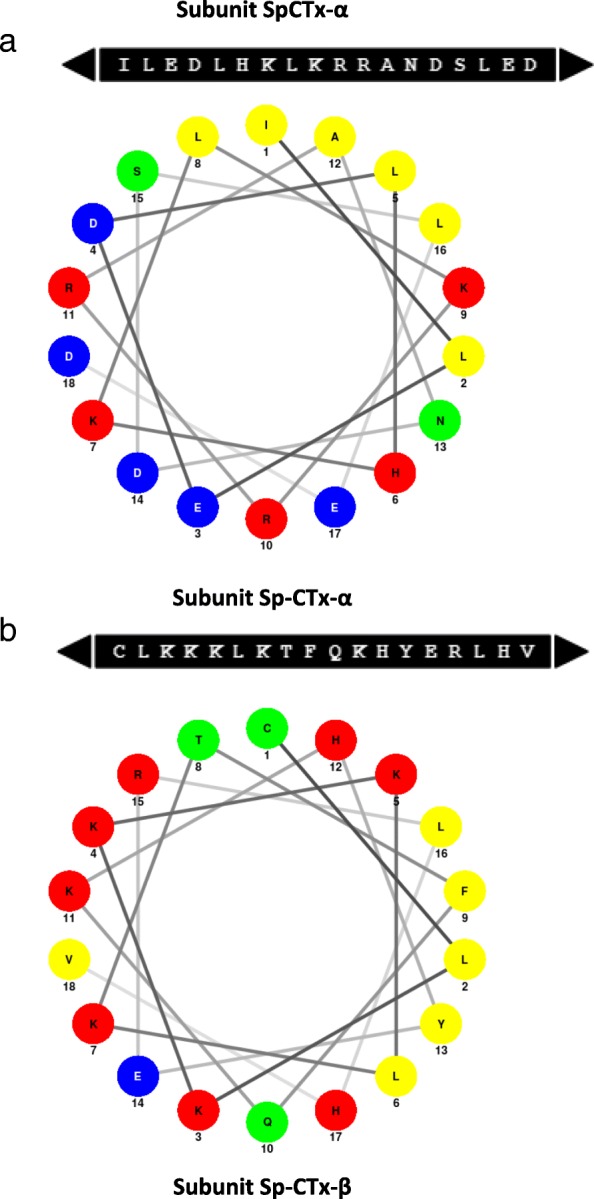
Predicted amphiphilic α-helices in Sp-CTx α- and β-subunits. Two potential amphiphilic α-helices were predicted by Helical Wheel Projections by Schiffer-Edmundson [39, 40]. Residues are colored according to their chemical character as follows: acidic (blue), basic (red), uncharged polar (green) and nonpolar (yellow). **a** Amphiphilic α-helix from Ile275 to Asp292 in Sp-CTx-α subunit; **b** Amphiphilic α-helix from Cys300 to Val311 in the Sp-CTx-β subunit

The prediction of secondary structures in Sp-CTxs (PSIPRED) posits the presence of five amphiphilic α-helices with a minimum size of 20 amino-acid residues (three in α- and two in β-subunit). Applying the “Edmunson Wheel” diagram, some predicted helixes exhibited cytolytic potential, as the hydrophobic portion is concentrated opposite to the hydrophilic side, revealing its amphiphilicity (Fig. 4). For instance, one amphiphilic α-helix was predicted between Gln_266_ and Asp_292_ in Sp-CTx-α and the diagram design shows the final 18 residues starting with Ile_275_ that supports the potential of this domain, as shown in Fig. 4a with the upper hydrophobic residues (Leu_282_, Ile_275_, Ala_286_, Leu_279_ and Leu_290_). Interestingly, the N-terminal of this helix is flanked by a region that contains basic residues (Arg_270_, Lys_271_ and His_273_), providing the cationic site common to proteins displaying cytolytic activity. Another α-helix with cytolytic potential was predicted between Cys_300_ and Val_317_ in Sp-CTx-β. The presence of amphiphilic residues Ser_294_ and His_311_ and the N-terminal flanking residues Lys_302_, Lys_303_, Lys_304_ and Lys_306_ support the cytolytic feature assigned to this domain (Fig. 4b).

### Comparative modeling

**Fig. 5:**
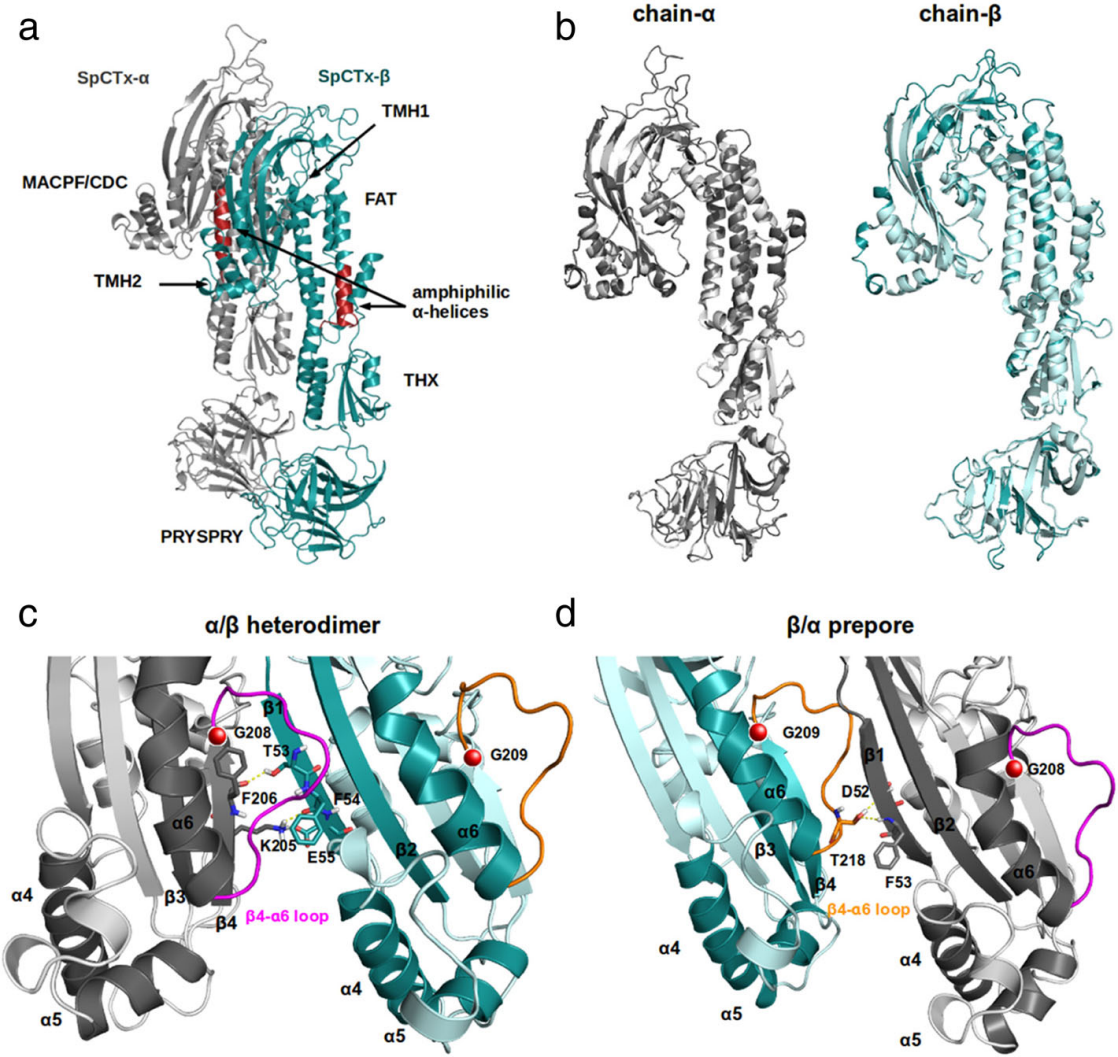
Three-dimensional modeled structure of Sp-CTx. The Sp-CTX modeled structures are shown in cartoon format. **a** Modeled structure showing interactions between Sp-CTx subunits; Sp-CTx-α (gray) and Sp-CTx-β (blue). Identification of the N-terminal domains; MACPF/CDC, FAT, THX and PRYSPRY, the transmembrane α-helices TMH1 and TMH2, the amphiphilic α-helices (red) of Sp-CTx-α and β-chains. **b** The structure of Sp-CTx aligned with SNTX (Protein Data Bank ID code 4WVM) and schematic representation of α-subunits (gray) on the left and β-subunits (blue) on the right. Lighter tones depict the structure of SNTX. **c** Highlighted interface region within the heterodimer in the MACPF/CDC with β-strands numbered according to their position in the central β-sheet. The β4-α6 loop is shown in pink, the conserved G208 (Sp-CTx-α) is shown as a red sphere. Hydrogen bonds between the residues F206 and K207 of strand-β4 (Sp-CTx-α) and T53, F54, E55 of strand-β1 (Sp-CTx-β) are displayed as yellow dashed lines. **d** The interface region of prepore in MACPF/CDC. The β4-α6 loop is colored orange, the conserved G209 (Sp-CTx-β) is shown as a red sphere. Hydrogen bonds between residues T218 in β4- α6 loop (Sp-CTx-β) and D52, T53 of strand-β1 (Sp-CTx-α) are shown as yellow dashed lines. Figures were generated using Pymol (v1.7.0.0), (http://www.pymol.org/; Delano Scientific LLC, South San Francisco, CA)

Using BLAST and Protein Data Bank tools, we found 55% and 68% sequence identity between α-subunit and β-subunit of Sp-CTx and venom homologues in SNTX (*S. horrida*), considered sufficient to infer structural conservation (Table 2). The structure of the latter was determined by X-ray crystallography at the resolution of 3.1 Å (PDB: 4WVMA and 4WVMB, chains α and β). Despite its moderate resolution, SNTX was used as the template since it is the only structure available for this toxin in Scorpaeniformes. The automated mode of SWISS-MODEL was used for template identification, alignment and generation of the models. Using each subunit from Sp-CTx, a single model was built by the server followed by Ramachandran plot, ProSA and QMEAN analysis for model validation (Fig. 5a).

Ramachadran Plot analysis of Sp-CTx model allocated 93.2–94.5% of amino-acid residues in favored regions, 4.2–5.8% in allowed regions and 1.0–1.3% in disallowed positions, confirming the stereochemical quality of the model. The ProSA server was used for evaluating potential errors of the models. The overall quality for the Sp-CTx-α model, expressed as the z-score was − 11.71, while the z-score for the SNTX-α template was − 9.82. The predicted z-score for the β-subunit was − 11.85, meanwhile the template z-score was − 10.04. For both models (α and β) the predicted z-scores for Sp-CTx are within the range observed for experimentally determined SNTX structures.

The QMEAN z-score for quality of Sp-CTx-α was − 3.23, and − 2.57 for SNTX-α. The QMEAN score for Sp-CTx-β was − 2.87 and for the subunit β of the template was − 2.05. Although the z-scores for Sp-CTxα-β are far from zero, they are within the range of values calculated for the respective template. According to QMEAN, the predicted differences between the models and the crystallographic structure are mainly due to changes of torsion angles exhibiting respective z-scores of 2.94 and − 2.57 for α- and β-subunit in Sp-CTx, while z-scores were − 2.46 and − 1.92 in α- and β-subunits from SNTX.

The modeled structures of SNTX and Sp-CTx were superimposed when RMSD in backbone atoms were 0.170 Å and 0.142 Å, for α- and β-subunits, respectively (Fig. 5b). These low RMSD values highlight the extensive superposition between the model and the template with minimum deviation from backbone atoms. We then predicted the structure of the heterodimer complex composed with the modeled subunits. For that purpose, interacting interface residues were predicted at the InterProSurf web server and possible binding modes were calculated using HADDOCK. The protocol identified via rigid body docking, semi-flexible docking, and explicit solvent refinement 398 complex structures grouped into 5 clusters. According to HADDOCK protocol cluster 2 was the most reliable, encompassing 78 members and exhibiting a z-score of − 1.2 (a more negative value is considered better, while remaining clusters had z-scores between − 0.8 and 1.5). Each complex from cluster 2 was superposed with the crystallographic structure (PDB ID: 4WVM) and their respective RMSD calculated. The structure with the lowest RMSD (1.1 Å), calculated from the backbone atoms was selected for further analysis.

The 3D structure shows (Fig. 5a) that Sp-CTx-α and -β form a dimer containing a mixture of α/β folds comprising four distinct domains: a MAPCPF/CDC domain, a focal adhesion-targeting (FAT) motif, thioredoxin (THX), and finally, the C-terminal domain containing PRYSPRY. A predicted secondary amphiphilic α-helix is shown (red) in Fig. 4 within the FAT domain.

The interface between α- and β-subunits of Sp-CTx has many features contained in the SNTX-α/β heterodimer. Figure 5b reveals that both toxins present strong structural similarity within each heterodimer. A highly conserved loop was found in the interface between subunits. In Sp-CTx, the β4-α6 binding site contains a hydrophobic surface comprising TMH2, helix-α6 and strand-β1, which is equivalent to MACPF and CDCs structures in SNTX, thus suggesting that this region is important for dimer formation, stability and oligomerization events (Fig. 5c, d). This analysis indicates that several noncovalent interactions stabilize the dimer interface in Sp-CTx.

### Phylogenetic study of Sp-CTx

**Fig. 6:**
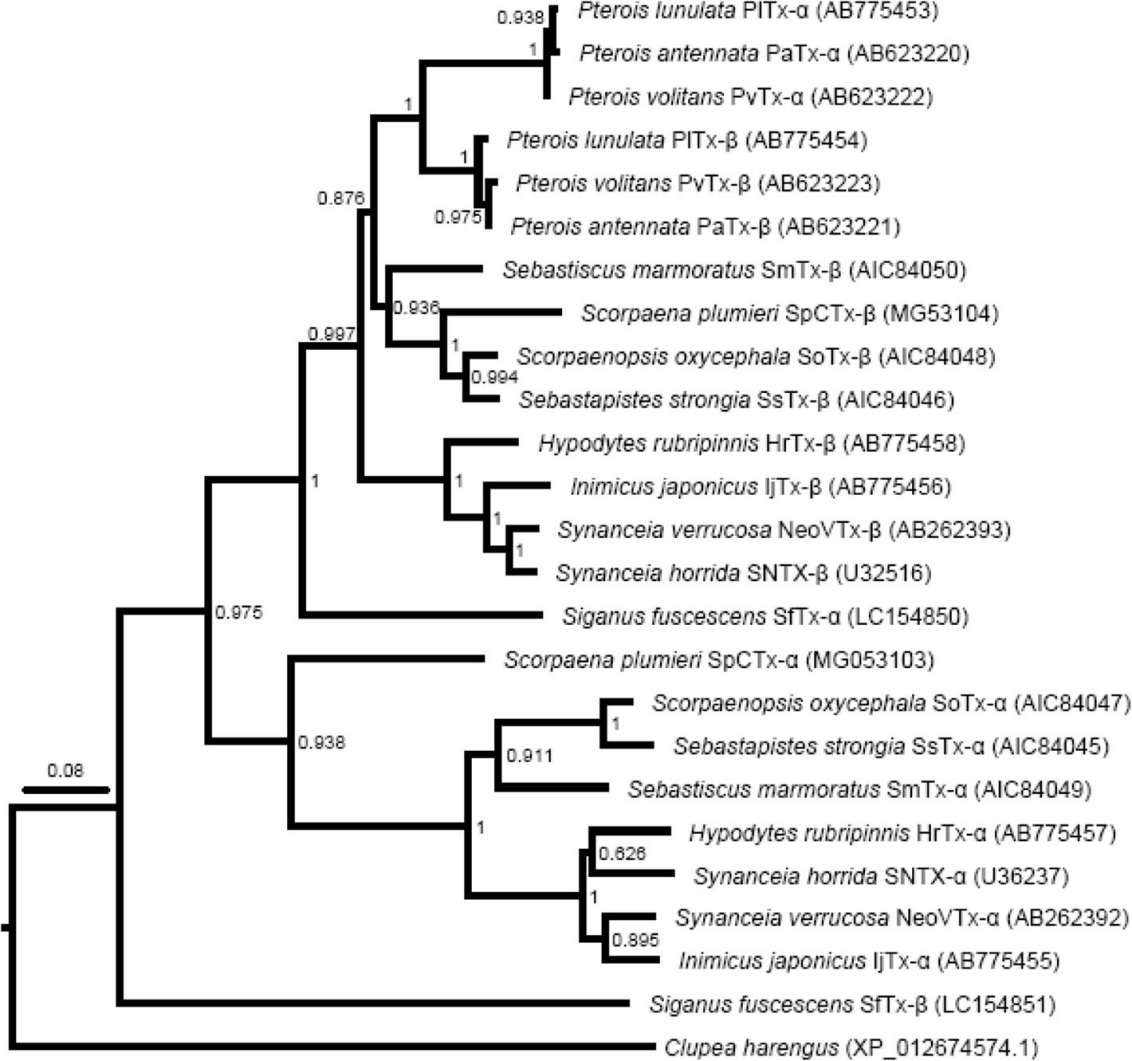
Phylogenetic tree of Scorpaeniformes toxins. The tree was generated by the MUSCLE [42] and then analyzed with the Neighbor-Joining algorithm (bootstrap replicates: 500; substitution model: Maximum Composite Likelihood), both implemented in MEGA7 [43]. The selected sequences (DDBJ/EMBL/GenBank nucleotide databases) and the accession numbers are: AIC84049 and AIC84050 (*Sebastiscus marmoratus*), AIC84047 and AIC84048 (*Scorpaenopsis oxycephala*), AIC84045 and AIC84046 (*Sebastapistes strongia*), AB775453 and AB775454 (*Pterois lunulata*), AB623222 and AB623223 (*Pterois volitans*), AB623220 and AB623221 (*Pterois antennata*), AB775455 and AB775456 (*Inimicus japonicus*), AB775457 and AB775458 (*Hypodytes rubripinnis*), AB262392 and AB262393 (*Synanceia verrucosa*) and U36237 and U32516 (*Synanceia horrida*), LC154850 and LC154851 (*Siganus fuscescens*). As an outgroup, we included in the analysis a stonustoxin subunit beta-like protein sequence from *Clupea harengus* (accession number: XP_012674574.1)

The phylogenetic tree of Sp-CTx is shown in Fig. 6. Accordingly, toxins were grouped into three distinct clusters: i) *Pterois* sp. and Subunits β group: PlTx-α, PaTx-α, PvTx-α, PlTx-β, PvTx-β and PaTx-β from *Pterois lunulata*, *Pterois antennata*, *Pterois volitans*, *Pterois lunulata*, *Pterois volitans* and *Pterois antennata* respectively; Subunit β group: SmTx-β, Sp-CTx-β, SoTx-β, SsTx-β, HrTx-β, IjTx-β, NeoVTX-β and SNTX-β from *Sebastiscus marmoratus*, *Scorpaena plumieri*, *Scorpaenopsis oxycephala*, *Sebastapistes strongia*, *Hypodytes rubripinnis*, *Inimicus japonicus*, *Synanceia verrucosa* and *Synanceia horrida*, respectively; in addition SfTx-α is classified in the same cluster despite its apparent differences with other members; ii) Subunits of the α group: Sp-CTx-α, SoTx-α, SsTx-α, SmTx-α, HrTx-α, SNTX-α, NeoVTX-α and IjTx-α from *S. plumieri*, *Scorpaenopsis oxycephala*, *Sebastapistes strongia*, *Sebastiscus marmoratus*, *Hypodytes rubripinnis*, *Synanceia horrida*, *Synanceia verrucosa* and *Inimicus japonicus*, respectively; and iii) finally, β-subunit from Perciforme *Siganus fuscescens* included in a separate cluster. The phylogenetic analysis suggests that genes coding for subunits from all species belong to two different clusters (β and α clades) except for *Pterois*, whose subunits are grouped together and branch out from the first clade.

## Discussion

A pore-forming cytolysin from *S. plumieri* venom (Sp-CTx) that induces cardiovascular alterations and other pharmacological activities has been purified by [11, 16]. Pharmacological effects similar to Sp-CTx have been attributed to other hemolytic factors from stonefish venoms [19, 20, 47, 48]. The cardiovascular effect induced by Sp-CTx is observable both in vitro and in vivo, and includes a vasorelaxant action that appears to involve the L-arginine-nitric oxide synthase pathway [16]. It has been suggested that the cardiovascular effect of Sp-CTx is caused by increased influx of sarcolemma Ca^2+^ affecting ventricular cardiomyocytes [22].

The structural features accounting for the pharmacological properties of Sp-CTx are poorly defined mainly because of the limited amounts available in fish venom [4]. To gain insight into the venom protein composition, we initially produced a cDNA library from *S. plumieri* to prospect by random EST the major gland components. While several lectins were identified in spine tissue, none of the readouts provided information on Sp-CTx [28].

Identification of Sp-CTx was then attempted using the library with two primers (β_T_-f and CDβ-r) whose sequences were derived from Scorpaeniformes toxins. The sequenced fragment annealed to β toxins of three families already identified as lethal factors and covering 74% of the β-subunit. Attempts to recover the missing 26% region in the library were unsuccessful. Instead, the missing C-terminal complement of Sp-CTx-β was identified in the total cDNA fraction from *S. plumieri* with CDβr-f primers.

For Sp-CTx-α subunit, the entire sequence was identified and assembled following amplification of four overlapping segments from the same cDNA fraction (Fig. 1). The initiators for isolation of Sp-CTx-α were derived from conserved nucleotide sequences reported in lionfish and stonefish toxins. The deduced sequences (Sp-CTx-α and Sp-CTx-β) from *S. plumieri* fulfill the prospects of lethal factors described in Scorpaeniformes. The deduced ORFs encode two polypeptides encompassing 702 amino-acids each and predicted mass of 80,153 kDa for Sp-CTx-α and 79,816 for Sp-CTx-β. The predicted mass for Sp-CTx-α and -β subunits resemble those of cytolysins identified in Scorpaeniformes venoms [13–15, 19, 20].

Gomes et al. [11], estimated the size of Sp-CTx complex to be 150 kDa based on non-reducing and denaturing electrophoretic evidence, in agreement with the figure deduced herein for Sp-CTx-α + Sp-CTx-β. The authors also identified internal peptides in Sp-CTx by Orbitrap-MS analysis of the trypsinized purified protein. Eight fragments totaling 79 residues (11.2%) were identified in Sp-CTx-α, whereas twelve fragments totaling 116 residues (16.5%) were identified in Sp-CTx-β, matching the sequences found herein, as shown in Fig. 2a, b (fragments highlighted in boxes). A search using SignalP 4.0 tool did not detect a signal peptide-like motifs in either Sp-CTx-α or β-subunit, similarly to other Scorpaeniform toxins described to date [19, 49]. The absence of muscular tissue in venom glands indicates that mechanical pressure is required to release the venom through the spinal system [8]. An interesting feature in Scorpaeniformes toxins is the presence of a B30.2/SPRY domain in their C-terminal regions. This domain is also found in diverse protein families, such as TRIM (Tripartite motif), RBCC (RING-finger, B-box plus coiled-coil domain), BTN (butyrophilin) and SPSB (cytokine signaling box protein) [50]. This highly variable domain possibly recognizes a specific protein ligand [51]. The functional role of B30.2 and SPRY domains is unclear, although it is evolutionarily preserved. It displays three conserved motifs, containing LDP, WEVE and LDYE [50, 52]. The LDP motif is identifiable in Sp-CTx-α at position 527–529, the WEVE motif is found both in Sp-CTx-α and -β at positions 578–581 and 579–582, respectively, and the LDYE motif is absent in both subunits. On the other hand, the crystal structure of SNTX reveals that the PRYSPRY domains in the heterodimeric toxin located distally to the N-terminal end are structurally similar to protein domains involved in innate immunity against microorganism infection. The mediation of its action by protein-protein and protein-lipid interactions on the cell surface suggests a mechanism for toxicity in SNTX [10]. A comparative structural analysis between Sp-CTx and SNTX crystals revealed the presence of three shared domains: Membrane Attack Complex-Perforin/Cholesterol-Dependent Cytolysin (MACPF/CDC), focal adhesion-targeting (FAT) and thioredoxin (THX), [10].

MACPF/CDC proteins are perforins found in diverse organisms typically composing a ring-shaped supramolecular oligomeric membrane pore complex, such as in pathogenic gram-positive bacteria and in the mammalian complement immune system [53]. This domain interacts with FAT, which has a signaling function [54], and a region structurally similar to mitochondrial thioredoxin (THX) from *Saccharomyces cerevisiae*. However, the THX domain is not involved in redox reactions because it lacks a catalytic site [55].

Three-dimensional modeling was necessary because the alignment of primary sequences was insufficient to analyze the spatial orientation of Sp-CTx residues and their molecular interactions. By building the model for each subunit and obtaining the predicted heterodimer by docking, we were able to examine in detail the heterodimer interface and to identify interactions that stabilize it.

The data led us to propose that Sp-CTx also belongs to the pore-forming MACPF/CDC superfamily, sharing a common four-strand folding and a highly twisted β-sheet anchored to three small α-helix clusters, in which two of these helical regions insert into the membrane (transmembrane hairpins TMH1 and TMH2). Interestingly, the structural folding of these domains resembles the crystallographic structures of other proteins, such as those responsible for protein-cell interactions occurring during immunological recognition [52]. Previous studies show that when pores are formed by CDCs, the monomers assemble into a prepore unit on the membrane surface and that the ensuing pore formation involves significant secondary and tertiary structural changes in TMH1 and TMH2 to penetrate the membrane as amphipathic β-hairpins [56].

Similar to stonefish toxins, Sp-CTx displays 50% identity between its α- and β-subunits, while lionfish toxins are approximately 80% identical. It is unclear whether these variations in identities between subunits in stonefish and lionfish are related to species-specific functions. Because of this strong identity [19], it was proposed that SNTX genes for α- and β-subunits evolved separately from a common ancestor after gene duplication.

A similarity search between Sp-CTx-α or Sp-CTx-β and similar annotated sequences using the NCBI database and BLAST algorithm [41] revealed significant identity only with toxins from Scorpaeniformes. Five cysteinyl residues located at similar positions in described subunits appear to be involved in protein conformation through disulfide bridges. Ghadessy and cols. [19] identified, by titration of SNTX with DTNB, five free cysteines and ten cysteines involved in intrachain disulfide bridges. However, in Sp-CTx these residues did not interact in the heterodimer model.

Different from toxins in terrestrial animals displaying toxin isoforms encoded by more than two alleles, there is no information to indicate the number of copies in fish toxins. Chuang and Shiao, [15] suggested that gene duplication occurred in the mother species of Scorpaeniformes where they evolved into α and β subunits. The authors identified an additional toxin duplication that can be found as a pseudogene in the lineage of lionfish.

Cationic residues like lysine and arginine and the hydrophobic amino acid tryptophan are essential for the cytolytic activity in toxins [44, 57]. The membrane-permeating ability of many peptides and proteins can be attributed to the presence of hydrophobic segments or amphiphilic α-helices and β-sheets [44]. Chuang and Shiao, [15] reported 23 positively-charged amino acids and 6 conserved tryptophanyl residues in every Scorpaeniformes toxin described, a rule that is confirmed in Sp-CTx. Additional studies by site-directed mutagenesis would be useful for clarifying the role of these residues.

To investigate the evolutionary relationships among Scorpaeniformes toxins, a phylogenetic tree was constructed and is displayed in Fig. 6. The classification of Sp-CTx agrees with previous evolutionary trees involving lethal factors [14, 15, 17]. In the diagram the amino-acid sequence of Sp-CTx is closest to those of scorpionfish and lionfish toxins followed by waspfish, stonefish and devil stinger toxins. Interestingly, the phylogenetic tree is consistent with the taxonomic classification based on the morphology of the venom glands described by Russell [58] and Halstead [1] who classified Scorpaeniformes into lionfish (*Pterois*) with shorter spines, scorpionfish (*Scorpaena*) with moderate spines and stonefish (*Synanceia*) with longer spines and highly developed tissue glands. The calculated sequence identities are reflected in the phylogenetic tree in which *Siganus fuscescens* toxin branches out from members of Scorpaeniformes, especially for β-subunit [17].

## Conclusion

In this study we identified the putative sequences coding for Sp-CTx, a lethal cytolysin from *S. plumieri* whose biochemical properties and pharmacological actions had been previously characterized. By comparative modeling with the SNTX structure, we identified potential determinants in Sp-CTx responsible for the cytolytic activity demonstrated in this toxin. The modeled Sp-CTxα-β heterodimer fits appropriately with the structure of SNTX from *S. horrida* identified by crystallography, thus supporting the notion that these proteins share similar functions.

## Additional file

Additional file 1: Alignment of the amino-acid sequences of Scorpaeniformes toxins. α-subunits of Sp-CTx, Sm-Tx, So-Tx, Ss-Tx, Pl-Tx, Pv-Tx, Pa-Tx, Hr-Tx, neoVTX, SNTX and Ij-Tx. β-subunits of Sp-CTx, Sm-Tx, So-Tx, Ss-Tx, Pl-Tx, Pv-Tx, Pa-Tx, Hr-Tx, neoVTX, SNTX and Ij-Tx. Single-letter amino-acid notation is used. Cysteine residues are highlighted in white on a black background. Identical residues are identified by a segment; (*) denotes conserved cationic residues; (\) denotes conserved tryptophan. Accession numbers (DDBJ/EMBL/GenBank nucleotide sequence databases): α- and β-subunits: Sm-Tx from *Sebastiscus marmoratus* toxin, AIC84049 and AIC84050; So-Tx from *Scorpaenopsis oxycephala* toxin, AIC84047 and AIC84048; Ss-Tx from *Sebastapistes strongia* toxin, AIC84045 and AIC84046; Pl-Tx from *Pterois lunulata* toxin, AB775453 and AB775454; Pv-Tx from *Pterois volitans* toxin, AB623222 and AB623223; Pa-Tx from *Pterois antennata* toxin, AB623220 and AB623221; Ij-Tx from *Inimicus japonicus* toxin, AB775455 and AB775456; Hr-Tx from *Hypodytes rubripinnis* toxin, AB775457 and AB775458; neoVTX from *Synanceia verrucosa*, AB262392 and AB262393 and SNTX from *Synanceia horrida*, U36237 and U32516. (DOCX 176 kb)

## Abbreviation

Sp-CTx: *Scorpaena plumieri* Cytolytic Toxin
