# Supplementary material for: Sequence analysis of the cDNA encoding for SpCTx: a lethal factor from scorpionfish venom (Scorpaena plumieri)
Source: J Venom Anim Toxins Incl Trop Dis. 2018 Aug 29;24:24. doi: 10.1186/s40409-018-0158-7 (PMC6114736; doi:10.1186/s40409-018-0158-7)
Supplement: Supplementary file 1 [file 40409_2018_158_MOESM1_ESM.docx]

```
SpCTx-a   M S S D I - I M A G L G R P F T L G F L Y D A R R E K L I P G F S L F G D E T L Q K Y A T S T P Q H S S D F Q I V A S D S V E S K S N V M D I E A S L G V S F L
SmTx-a    . . . . . M V . . . . . . . . . . . M . . . . . . . . . . . . . . M . . . . . . . Q . . S . N . . R . . . . . . . . . . . I N D . . S L . . . . . . . . . . . .
SoTx-a    . . . . . M . . . . . . . . . . . . M . . . . . . . . . . . . . . . . . . . . . . Q . . . . . . . R . . E . . . . . . . . . . . . . . L . . . . . . . . . . . .
SsTx-a    . . . . . M V . . . . . . . . . . . M . . . . . . . . . . . . . . . . . . . . . . Q . . . . . . . R . . E . . . . . . . . . . . . . . L . . . . . . . . . . . .
PlTx-a    . . . E . L . V . A . . . . . . . . A . . . S . K D R . . . . . T . W E . D V . . A N T V E S R . P . . A . E . I . . . . I . D . . S L R . . . . . . K A . . .
PvTx-a    . . . E . L . V . A . . . . . . . . A . . . S . K D R . . . . . T . W E . D V . . A N T V E S R . P . . A . E . I . . . . I . D . . S L . . . . . . . K A . . .
PaTx-a    . . . E . L . V . A . . . . . . . . A . . . S . K D R . . . . . T . W E . D V . . A N T V E S R . P . . A . E . I . . . . I . D . . S L . . . . . . . K A . . .
HrTx-a    . . . . . M . . P . . . . . . . . . M . . . . . K . . . . . . . . . . . . . . . . Q . Q S . N A . R . . E . . . . . . . . . T . . . . S A . . . . . . . . . . . .
NeoVTx-a  . . . . - L V . P A . . . . . . . . M . . . T . . . . . . . . . . . . . . . . . . Q . Q S . N T . R . . E . K . . . . . . T . . . . S A . . . . . . . . . . . .
SNTX-a    . . . . - L V . P A . . . . . . . . M . . . . . . . . . . . . . . . . . . . . . . . . Q S . N A . R . . E . K . . . . . . T . . . . S A . . . . . . . . . . . .
IjTx-a    - M . . . M V . P A . . . . . . . . M . . . T . K . . . . . . . . . . . . . . . . Q . E S . N A . R . . E . K . . . . . . T . . . . S A . . . . . . . . . . . .
SpCTx-b   . P . . . L V V . A . . . . . . . . A . . . . . K D . . Y . . . T . W E H . V . E E S T V E S D . P . . T . E . T . . . . I D D . . S L . . . . . . . K A . . .
SmTx-b    . . . E . L V V . A . . . . . . . . T . . . . . K D . . . . . . T . W D . . V . . E S T V E S C . P . . A . E . I . . . . I D D . . S L . . . . . . . K A . . .
SoTx-b    . . . . . L V L . A . . . . . . . . T . . . . . K D . . . . . . T . W E . . V . . E N T V E S A . P . . V . E . T . . . . I . D . . S L . . . . . . . K A . . .
SsTx-b    . . . . . L V V . A . . . . . . . . T . . . . . K D . . . . . . T . W E . . V . . E N T V E S A . P . . V . E . T . . . . I . D . . S L . . . . . . . K A . . .
PlTx-b    . . . Q . L V V . A . . . . . . . . T . . . . . K D R . . . . . T . W E . D V . . A N T V E S R . P . . A . E . I . . . . I . D . . S L . . . . . . . K A . . .
PvTx-b    . . . Q . L V V . A . . . . . . . . T . . . . . K V R . . . . . T . W E . D V . . A N T V E S R . P . . A . E . I . . . . I . D . . S L . . . . . . . K A . . .
PaTx-b    . . . Q . L V V . A . . . . . . . . T . . . . . K D R . . . . . T . W E . D V . . A N T V E S R . P . . A . E . I . . . . I . D . . S L . . . . . . . K A . . .
HrTx-b    . . . . . L V V P A . . . . . S . . A . . . . . K D . . V . . . T . W D . D V . E E S T V E S S . P . . A . E . I . . . . I D D . . S L . . . . . . . K A . . .
NeoVTx-b  . P . . . L V V . A . . . . . . . . M . . . . . N D . . . . . . T . W E . . V I E E S T V E S S . P . . A . E . I . . . . I D D . . S L . . . . . . . K A . . .
SNTX-b    . P . . . L V V . A . . . . . . . . M . . . . . N D . . . . . . T . W E . . V I E E S T L E S S . P . . A . E . I . . . . T D D . . S L . . . . . . . K A . . .
IjTx-b    . P . . . L V V . A . . . . . . . . M . . . . . N D . . . . . . T . W D . . V . D D E S T V E S S . P . . A . E . I . . . . I D D . . F L . . . . . . . E A . . .
                                  *                     *     *                                                                           *

SpCTx-a   G G L V E V G G S A K Y L N N T K K Y Q N Q S R V T L Q Y K V T T T F K Q F K A P P G K V N V Q Q T A I S D K N V A T H V V T A I L F G A N A F F V F D S D K V
SmTx-a    . . . . . . . . . . . . . . . . . . . . . . . . . . . K . . A . . . Y Q . . T . . . . T . K . . . . . . T E . G L . . . . . . G . . Y . . . . . . . . . . . . L
SoTx-a    . . . . . . . . . . . . . . . . . . . . . . . . . . . K . . . . . . Y Q . . . . . . . . . . . . . . . . T . . D . . . . . . . . . . . . . . . . . . . . . G . L
SsTx-a    . . . . . . . . . . . . . . . . . . . . . . . . . . . K . . . . . . Y Q . . . . . . . . . . . . . . . . . . . . . . . . . . . . . . . . . . . . . . . . . . . L
PlTx-a    . . . . . . A . . . . . . D D S . . F K . . . . . . . . . . A . . S . . . L M T N L E T K H . E Y S E Y F Q N L E . . . . . I G . . Y . . . . . . . . . . A . T
PvTx-a    . . . . . . . . . . . . . D D R . . F K . . . . . . . . . . A . . S . . . L M T N L E T K H . E Y S E Y F Q N L E . . . . . I G . . Y . . . . . . . . . . A . T
PaTx-a    . . . . . . A . . . . . . D D S . . F K . . . . . . . . . . A . . S . . . L M T N L E T K H . E Y S E Y F Q N L E . . . . . I G . . Y . . . . . . . . . . A . T
HrTx-a    . . . . . . . . . . . . . . . N . . . . . . . . . . . K . . S . . I Y . . . T . . . . T . K . . E . . . T E . G L . . . . . . S . . Y . . . . . . . . . . . . .
NeoVTx-a  . . . . . . . . . . . . . . . . . . . . . . . . . . . K . . A . . I Y . . . T . . . . T . K . . E . V . T Q R G L . . . . . . G . . Y . . . . . . . . . . . . .
SNTX-a    . . . . . . . . . . . . . . . . . . . . . . . . . . . K . . A . . V Y . . . T . . . . T . T . . E . . . T E . G L . . . . . . S . . Y . . . . . . . S . . . . .
IjTx-a    . . . . . . . . . . . . . . . . . . F . . . . . . . . K F . A . . V Y . . . T . . . . T . K . . E . V . T Q R G L . . . . . . G . . Y . . . . . . . . . . . . .
SpCTx-b   . . . I . . . . . . . . . . D . . . F K . . . . . . . . . . A . . S . . . L M T N L E T K H . E Y S E Y F Q N I E . . . . . I G . . Y . . . . . . . . . . . . .
SmTx-b    . . . . . . . . . . . . . . D . . . F K . . . . . . . . . . A . . S . . . L M T H L . T K H . E Y S E Y F Q N I E . . . . . I G . . Y . . . . . . . . . . . . I
SoTx-b    . . . . . . . . . . . . . . D . . . F K . . . . . . . . . . A . . S . . . L M T N L E T K H . E Y S E Y F Q N I E . . . . . I G . . Y . . . . . . . . . . . . .
SsTx-b    . . . . . . . . . . . . . . D . . . F K . . . . . . . . . . A . . S . . . L M T N L E T K H . E Y S E Y F Q N I E . . . . . I G . . Y . . . . . . . . . . . . .
PlTx-b    . . . . . . . . . . . . . D D R . . S K . . . . . . . . . . A . . S . . . L M T N L E T K H . E Y S E Y F Q N L E . . . . . I G . . Y . . . . . . . . . . A . T
PvTx-b    . . . . . . . . . . . . . D D R . . F K . . . . . . . . . . A . . S . . . L M T N L E T K H . E Y S E Y F Q N L E . . . . F I G . . Y . . . . . . . . . . A . T
PaTx-b    . . . . . . . . . . . . . D D R . . F K . . . . . . . . . . A . . S . . . L T T N L E T K H . E Y S E Y F Q N L E . . . . . I G . . Y . . . . . . . . . . A . T
HrTx-b    . . . . . . . . . . . . . . . Q . . F K . . . . . . . . . . A . . S . . . L M T N L . T K H . E Y S E L F E N I Q . . . . . I G . . Y . . . . . . . . . . N . .
NeoVTx-b  . . . . . . . . . . . . . . . Q . . F K . . . . . . . . . . A . . N . . . L M T N L . T K H . E Y S E L F E N I Q . . . . . I G . . Y . . . . . . . . . . N . .
SNTX-b    . . . . . . . . . . . . . . . Q . . F K . . . . . . . . . . A . . S . . . L M T N L . T K H . E Y S E L F E N I Q . . . . . I G . . Y . . . . . . . . . . N . .
IjTx-b    . . . . . . . . . . . . . . . Q . . F K . . . . . . . . . . A . . S . . . L M T N L . T K H . E Y S E L F . N I Q . . . . . I G . . Y . . . . . . . . . . N . .
                              *           * *           *           *                                                                                                 *

SpCTx-a   E D S N L Q D I Q G K M E A V I K K I P S V S I E G S G S V Q L T D E E K S L A S N L S C K F H G D F L L E S L P T T F E E A V M T Y Q K L P E L V G E E A S D
SmTx-a    . . . . . . . . E . R . . . . . R . . . T . . . . . Y . . . K . . . . . . . . . . . . . . . . . . . . . . . . . . . . . . D . . T . . . N . . G . L . . . G T N
SoTx-a    . . . . . . . . . . . . . . . . . . . . T . . . . . F . . . . . . . . . . . . . . . . . . . . . . . . . . . . . . . . . . D . . . . . . . . . . . L . . . . T .
SsTx-a    . . . . . . . . . . . . . . A . . . . . T . T . . . . . . . . . . . . . . . . T . . . . . . . . . . . . . . . . . . . . . D . . T . . K T . . . L . . . . T .
PlTx-a    . S . . V . . . . . S . . . A . . . . . . . E . S . Q . . . K . . S . D S A I T D R F . . . . . . . V . . P . N . . . . . D . . Q . . . Q . . Q M M . K . - - N
PvTx-a    . S . . V . . . . . S . . . A . . . . . . . E . S . Q . . . K . . S . D S A I T D R F . . . . . . . V . . P . N . . . . . D . . Q . . . Q . . Q M M . K . - - N
PaTx-a    . S . . V . . . . . S . . . A . . . . . . . E . S . Q . . . K . . S . D S A I T D R F . . . . . . . V . . P . N . . . . . D . . Q . . . Q . . Q M M . K . - - N
HrTx-a    . . T . . . . . . . . . . . . . . . . . T I . . . . . . . . . . . G . . . . . . . . . . . . . . . . . . . . . . . . . . . D . . K . . . T . . T . L . D D S A .
NeoVTx-a  . . T . . . . . . . . . . . . . . . . . T I . . . . . A . . . . . . . . . . . . . . . . . . . . . . . . . . . . . . . . . D . . T . . . T . . T . L . . D G A S
SNTX-a    . . T . . . . . . . . . . . A . . . . . T I . . . . . A . . . . . . . . . . . . . . . . . . . . . . . . . . . . . . . . . D . . K . . . T . . T . I . . D G A N
IjTx-a    . . T . . . . . . . . . . . . . . . . . T I . . . . . A . . . . . . . . . . . . . . . . . . . . . . . . . . . . . . . . . D . . N . . . T . . T . L . . D G A S
SpCTx-b   D S . . V . . . . . S . . . . . . . . . . . E . S . Q . . . . . . S . . S D I T N S F . . . . . . . . H . P . N . . . . . D . . K . . . Q . . Q M M . K . - - T
SmTx-b    D S . . V . . . . . S . . . . . . . . . . . E . A . R . A . . . . S . . S D I T N S F . . . . . . . . F . T . N . . . . . D . . Q . . . Q . . Q M M . K . - - N
SoTx-b    D S . K V . . . . . S . . . . . . . . . . . E . S . Q . A . K . . S . . S D I T N S F . . . . . . . . H . T . N . . . . . D . . K . . . Q . . Q M M . K . - - T
SsTx-b    D S . . V . . . . . S . . . . . . . . . . . E . S . Q . . . K . . S . D S E V T N S F . . . . . . . . H . P . N . . . . . D . . K . . H Q . . Q M M . K . - - T
PlTx-b    . S . . V . . . . . S . . . A . . . . . . . E . S . Q . . . K . . S . D S A I T D G F . . . . . . . . F . P . N . . . . . D . . Q . . . Q F . Q M M . K . - - N
PvTx-b    . S . . V . . . . . S . . . A . . . . . . . E . S . K . E . K . . S . D S A I T . G F . . . . . . . . F . P . N . . . . . D . . Q . . . Q . . Q M M . K . - - N
PaTx-b    . S . . V . . . . . S . . . . . . . . . . . E . S . K . E . K . . S . D S A I T . G F . . . . . . . . F . P . N . . . . . D . . Q . . . Q . . Q M M . K . - - N
HrTx-b    D S T . V . E . . . Q . . . . . . . . . . . E . S . K . A . T . . S . . S D I T N G F . . E . . . . . F . T . N . . . . . D . . Q . . . Q . . Q M M . K . - - N
NeoVTx-b  D S T . V . E . . . Q . . . . . . . . . . . E . S . K A . . . . . S . . T D I T N S F . . E . . . . . F . T . N . . . . . D . . K . . . Q . . Q M M . K D - - N
SNTX-b    D S T . V . E . . . Q . . . . . . . . . . . E . S . K A . . . . . G . . T D I T N S F . . E . . . . . F . T T N . . . . . D . . K . . . Q . . Q M M . K D - - N
IjTx-b    D S T . V . E . . . Q . . . . . . . . . . . E M S . K A . . . . . S D . S D I T N R F . . E . . . . . F . T . N . . . . . D . . K . . . Q . . Q M M . K D - - N
                                          * *

SpCTx-a   G V P M K V W L V P L T R F Y S K A D L L V R D I S Q G L V R K V H S I L E D L H K L K R R A N D S L E D D T V K L - F P L L E K K L K N F Q K N Y S D - Y M T
SmTx-a    A . . . . . . . . . . K K . . . Q . . . . . . . . T V . G . . . I . . T . . E . . E . . . . C . E A M D . K L . . Q - L . . I H D R V S . . . Q L F Q . - . I L
SoTx-a    A . . . . . . . . . . K K . . . Q . . . . . . . . . . . I . . . . . . . . . . . . E Q R . . C . E A M D . R L . . Q - L . . I Y D R V S . . . Q I F Q . - . I L
SsTx-a    A . . . . . . . . . . R K . . . Q . . . . . . . . . . . I . . . . Q . . . . . . . E Q R . . C . E A I D . R L . . Q - L . . I H D R V S . . . Q L F Q . - . I L
PlTx-a    A . . . . . . . . . . V H . . . E . P K . M A . S . I S I L . R . R N T . . E M R E . E M . C . . A . . . N . . . . - . . . M V . . . N . . . N L F G V - . . S
PvTx-a    A . . . . . . . . . . V H . . . E . P K . M A . S . I S I L . R . R N T . . A M R E . E M . C . . A . . . N . . . . - . . . M V . . . N . . . N L F G V - . . S
PaTx-a    A . . . . . . . . . . V H . . . E . P K . M A . S . I S I L . R . R N T . . E M R E . E M . C . . A . . . N . . . . - . . . M V . . . N . . . N L F G V - . . S
HrTx-a    A . . . . . . . . . . N K . . . . S Q . . . . E . N V S K . . R I . T T . . E . . A . . . . . . E A M D . K L . Q R - I . . I H D . I S E . . Q I F Q . - . I L
NeoVTx-a  A . . . . . . . . . . K K . F . . . K . . T Q E . T V S K . . R I . T T . . E . Y . . . . . . . E A M D . K L . Q Q - I . . I H D . I S . . H Q I F Q . - . . L
SNTX-a    S . . . . . . . A . . K S Y N . . . Q Q . I Q E . N V S K . . R I . T T . . E . . . . . . . . . E A M D V K L . Q R - I . . I H D . I S . . . Q I F Q . - . . L
IjTx-a    A . . . . . . . . . . K K . . T . . Q . . I Q Q . . V S K . . R I . T T . . E . . . . . . . V . E A M D . K L . Q Q - . . . I H D . I S . . H Q I F Q . - . . L
SpCTx-b   A . . . T . . . . . M . N . . . E . P Q . M A . S . T P I L . . . R N T . . A M R Q . D M . C . . . . . R R H S E A G . H C . K . . . . T . . . H . E R L H V N
SmTx-b    A . . . T . . . . . . V H . . . E . P K . M A . S . S P I L . . . R N T . . A M R Q . E M . . . . . . . . . I . . . - . . . V R . . . S . . R . F C N . - . . L
SoTx-b    A . . . T . . . . . M . N . . . E . P Q . M A . S . T P I L . . . R N T . . A M R Q . D M . . . . . . . . . . . . . - . . . . . . . . . T . . . H . R . - . . .
SsTx-b    A . . . T . . . . . M . N . . . E . P Q . M A . S . T P V F . . . R N T . . A M R T . D M . . . . . . . . . . . . . - . . . . . . . . . T . . . H . R . - . . .
PlTx-b    A . . . T . . . . . . V N . . . E . P K . M A . S . T P I L . . . R N T . . A I R E . E M . . . . A . . . N . . . . - . . . I . . . . N . . K N F F G . - . . L
PvTx-b    A . . . T . . . . . . V N . . . E . P K . M A . S . T P I L . . . R N T . . A M R E . E M . . . . A . . . N . . . . - . . . I . . . . N . . K N F F G . - . . L
PaTx-b    A . . . T . . . . . . V N . . . E . P K . M A . S . T P I L . . . R N T . . A M R E . E M . . . . A . . . N . . . . - . . . I . . . . N . . K N F F G . - . . L
HrTx-b    A . . . T . . . . . M V N . . . E . P K . M A . S . T P V L . . . R N T . . A I V . V Q M . . . . A . D . P I . . . - . . Q V R . . . . S . . . N F C D . - H K L
NeoVTx-b  A . . . T . . . . . M V N . . . E . P Q . M A . S . T P I L . . . R N T . . A I V Q V Q M . . . . A . D . P . . N . - . T E V Q . . . S D . . I I C D . - H . S
SNTX-b    A . . . T . . . . . M V N . . . E . P Q . M A . S . T P I L . . . R N T . . A I V Q V Q M . . . . A . D . P . . N . - . T E V Q . . . S D . . . I C D . - H . S
IjTx-b    A . . V T . . . . . M S N . . . E . P Q . M A . S . T P I L . . . R D T . . A I V Q V Q M . . . . A . D . P I . N . - . T E V Q . . . S D . . . I C D . - H . S
                      \                                               * *                           *

SpCTx-a   T F R R T - I S Q K L Q S I R K G D E D E T A M L Q L F E D R L R S P F N I D S L N M W M E I T E R E I N V L R S C I D I I E E T K H K A V L S Q S Q M V K D L
SmTx-a    . V Q Q K - . A T . . P L . . G . T . . . S S L E K I I Q . . A K . . . S N E N V S T . L D A I . . . . T A . K A . A G M M . G . Q T . F . S N . T E L D R E V
SoTx-a    . V Q Q . - . A K . . P L . . E . T . E . S S L E E I I . . . A N . . . S N E N V . I . L D N I . . . . G S . K A . A G . L . K . Q T . F . S N . T E L D R E V
SsTx-a    . V Q Q . - . A K . . P L . . D . T . E . S S L E E I T . . . A N . . . S N E N V . I . L D N I . T . . G S . K A . A G . L . K . Q T . F . S N . T E L D R E V
PlTx-a    N L . . S - . A E . . . . . . S . S . . Q S . . . . . . Q E N . K . . . . T . . . . . . . D C A . . . . . . . . . . . . . . . . . . A R . . V . . . E . E . F R E .
PvTx-a    N L . . S - . A E . . . . . . S . S . . Q S . . . . . . Q E N . K . . . . T . . . . . . . D C A . . . . . . . . . . . . . . . . . . A R . . V . . . E . E . F R E .
PaTx-a    N L . . S - . A E . . . . . . S . S . . Q S . . . . . . Q E N . K . . . . T . . . . . . . D C A . . . . . . . . . . . . . . . . . . A R . . V . . . E . E . F R E V
HrTx-a    . V Q K K - . A E . . P L V . A . T . S . Q S L Q K I I D . . E K . . . S N E K A S A . L D A I . . . . A H . K . . A G M V . G . Q A . F . P N . T E L D . E V
NeoVTx-a  . V Q K K - . A E . . P L V . A . T . S . Q S L Q K I I D . . A K . . . S N E N V S T . L . V I . . . . A . . K . . A G M V . G . Q A . F . S N . T E L D R E V
SNTX-a    . V Q K K - . A E . . P L V . A . T . S . Q S L Q K I I D . . A Q . . . S N E K V S K . L D A V . . . . A . . K . . A G M V . G . Q A . F . S N . T E L D R E V
IjTx-a    . L Q K K - . A E . . P L V . A . T . S . Q S L Q K I I D E . A Q . . . S N E K V S . . L D A I . . . . A . . K T . A G M V . G . Q A . F . S N . T E L D . E V
SpCTx-b   P . . K N H F P E T F S P S G . . T K M K L Q C . P T . R N K . . . . S . . N . . . . . . D C A . . . . . . . . . . . . . . . . . . A . . . V . . . K . . . A R E .
SmTx-b    N L . G . - . A K . . . . . . S . G . . . S . . . K . . . Q N . . . . . . L . . . . . . . . C E . . . . . . . . . . V . . . . . . A . . . V . . . E N . . . R E .
SoTx-b    N L . . . - . . . . . . . . . . . . . . . S . . . . . . . . K . . . . . . . . . . . . . . . C A . . . . . . . . . . . . . . . . . . A . . . V . . . . T K L D R E .
SsTx-b    N . . . . - . . K . . . . . . . . . . . . S . . . . . . . . K . . . . . . T . . . . . . . . C A . . . . . . . . . . . . . . . . . . A . . . V . . . . . K . . R E .
PlTx-b    N L . . . - . A E . . . . . . S . S . . Q S . . . . . . Q E N . K . . . . . . . . . . . . D C A . . . . . . . . . . . . . . . . . . A R . . V . . . E . E . F R E .
PvTx-b    N L . . . - . A E . . . . . . S . S . N . S . . . . . . Q E N . K . . . . . . . . . . . . D C A . . . . . . . . . . . . . . . . . . A R . . V . . . E . E . F R E .
PaTx-b    N L . . . - . A E . . . . . . S . S . N . S . . . . . . Q E N . K . . . . . . . . . . . . D C A . . . . . . . . . . . . . . . . . . A . . . V . . . E . E V F R E .
HrTx-b    K L Q A . - . A K . . L A . . S . . . . . S . I . N V . . E N . Q . . . . . E . . . . Q . . D F E . . . . . . . . . . M . . L . . A . P . I . . . K . A . F . E .
NeoVTx-b  K L Q A . - . A K . . F A . . S . . . . . S . L V N . . . E N . Q . . . . . E . . . . . . . F E . . . . . . . K . . M . . L T K A . P . V I F N . G V L F . E .
SNTX-b    K L Q A . - . A K . . F A . . S . . . . . S . L . N . . . E N . Q . . . . . E . . . . . . . F E . . . . . . . . . . M . . L T K A . P . V I F N . G V L F . G .
IjTx-b    K L Q A . - . A K . . F A . . S . . . . . S . L . N . . . E N . Q . . . . P E . . . . Q . I A F E . . . . . . . . . . M . . L K K A . P . I . . N K N A I F . E .
                                                                                              \                                               *                   *

SpCTx-a   L D S E V M H A V C Y V F T Y V T D K D H Y L D A L R D Y L K S P N S R - P A R V R P V V T - - W V A S N T V L E T M R E K A H L F R S L A K D M E - N R C V H
SmTx-a    . A E N . K . . L . F . . . S M E R S . P . . R . . S E . . E . . . T E I T R D . A . S T E D M . C Y . T R . V L K . K Q . . Q Q . S L H . . K . . K S . N . V
```

```
SpCTx-a   L D S E V M H A V C Y V F T Y V T D K D H Y L D A L R D Y L K S P N S R - P A R V R P V V T - - W V A S N T V L E T M R E K A H L F R S L A K D M E - N R C V H
SmTx-a    . A E N . K . . L . F . . . S M E R S . P . . R . . S E . . E . . . T E I T R D . A . S T E D M . C Y . T R . V L K . K Q . . Q Q . S L H . . K . . K S . N . V
SoTx-a    . A E N . K . . L . F . . . . M E R N . P . . R V . S Q . . E . . D T E I T G D . A . S . E N - - - - N S R M V I K . K Q . . L A . S E F . . K L . K S . N . .
SsTx-a    . A E N . K . . L . F . . . . M E R N . P . . R V . A Q . . E . . D A E I . G N . A . S . E N - - - - N S R M V I K . K Q . . L D . S E F . . K L . K S . N . .
PlTx-a    . . . N . K . . . . . . . . F . . . Q . P F . K V . T . . . D . . D . . - . K . T . . P A . E Y . Y . . D . I P . M . . D . . . I . Y . . . . E . . - . . R . R
PvTx-a    . . . N . K . . . . . . . . F . . . Q . P F . K V . T . . . D . . D . . - . K . T . . P A . E Y . Y . . D . I P . M . . D . . . I . Y N . . . E . D - . . R . R
PaTx-a    . . . N . K . . . . . . . . F . . . Q . P F . K V . T . C . D . . D . . - . K . T . . P A . E Y . Y . . D . I P . M . . D . . . I . Y . . . E E . D - . . R . R
HrTx-a    . A E K . K . . L . F . . . S L E C I . P . . K V . S . . . E . . D . K D A T D . M . S I Q D M . F H . T E . V R K . K Q T . K T . F D H V N . F K K S . N . G
NeoVTx-a  . A E D . K . . L . F . . . S . E R N . P . . K V . S . . . E . . D . K D G K E A V . S T E D K . C F . T R . V L K . K Q R . Q T . C D H V N . F . K S . N . G
SNTX-a    . V G K . K . . . . F I . . S . E R N . P . . K V . S . . W E . . P . N N A K D . A . S T E D K . C F . T E . V L K . Q Q R . Q T . C D H V N . F . K S . N . G
IjTx-a    . A E D . K . G L . F . . . S . E R I . P . . K V . S . . . D . . D . K A A K D . S . S T E D K . C F . T G . V L K . K Q R . Q T . C D H V N E F . K S . N . G
SpCTx-b   D . . . . K . . . . . . . . . . . . Y . P F . N . . S . F - - . K S I K - . K K Y S . S K K D Y . Y T . D D . P . M . . . . . . H . Y N . . . . . . - . . . . R
SmTx-b    . . . K . Q . . . . . . . . . . . . C . P . . K V . S . F S D . . T . K - . K K F . . S T K D Y . Y . . D D . P . M . . . . . R . . Y N . . . . . D - . . . . R
SoTx-b    D . . . . K . . . . . I . . . . . . Y . P . . N . . S . F - - . K S I K - . K K F S . S K K D Y . Y T . D D . P . M . . . . . . H . Y N . . . . . . - . . . . R
SsTx-b    . . . . . K . . . . . . . . . . . . Y . P F . K . . S . F - - . K S I K - . K K Y . . S K K D Y . Y T . D D . P . M . . G . . . H . Y N . . . . . D - . . . . R
PlTx-b    . . P N . K . . . . . . . . F . . . Q . P F . N V . S . F . D . . T . K - . K K L . . S T K D Y . Y I . D D I P . M . . . . . . H . . N . . L . . D - . . . . R
PvTx-b    . . P N . K . . . . . . . . F . . . Q . P F . N V . S . F . D . . T . K - . K K L . . S T K D Y . Y I . D D I P . M . . A . . . H . . N . . L . . D - . . . . R
PaTx-b    . . P N . K . . . . . . . . F . . . Q . P F . N V . S . F . D . . T . K - . K K L . . S T K D Y . Y I . D D I P . M . . A . . . H . . N . . L . . D - . . . . R
HrTx-b    . . . K . K . G L . . . . . D . . . H . F F . N . . N . F S D . . Q . K - . K K L . . S P K E Y . Y S Y D D I P . M . . . . . R . . . N . S E E . N - . . . . R
NeoVTx-b  Y . . K . K . G L . . . . . N . . K N . D F . T V . N . F . D . . Q . . - . K K L . . S P K D Y . Y S Y D D I P . M . . . . . . . . . N . . . E . N - . . . . .
SNTX-b    Y . . K . K . . L . . . . . N . . K N . V F . N V . N E F . D . . Q . . - . K K L . . S P K D Y . Y S Y D D I P . . . . . . . Y . . . N . . . E . N - . . . . .
IjTx-b    Y . A K . K . G L . . . . . N . . N T . Y F . T V . N . F . D . . Q . K - . K K L . . S P K D Y . Y T Y D E I P . M . . . . . . . . C N . . . E . N - . . . . .
                                                                                                          \

SpCTx-a   F L V A S I V N L K V E G A A I H Y Y R E S V L I E P N F K H P I I S A V E K I V D R R D L L W Y D C E L T L D P - - N T S H P S L Y L S E G N K K A V T - - G
SmTx-a    . . A . A . T . G . Y Q . . S . Y H . K . G S . V T Q D . T F . . L V D A . R V K . . S . . . . . A . N . . F . . - - . . V . K C I T . . D D . . H . E S - - .
SoTx-a    . . A . A . T . G . Y Q . . S . Y H . K . G S . V T E D . T F . P L A P . K T . K . . S . . . . . A . D . . F . . - - . . A . Q Q . A . . D D . . Y V E S - - .
SsTx-a    . . A . A . T . G . Y Q . . S . Y H . K . G S . V T E D . T F . P M A P A K T . K . . S . . . . . A . D . . F . . - - . . A . Q V . A . . D D . . Y V E S - - .
PlTx-a    . . . . . . . . P . Q . . . G . . . . . . N I . . . Q E . L Q . K M N P . . . . . . . . . . . . . . . . . . . . . - - V . A . . A . T . . . . . . . . . S - - .
PvTx-a    . . . . . . . . P . Q . . . G . . . . . . N I . . . Q E . L Q . K M N P . . . . . . . . . . . . C . . . . . . . . - - V . A . . A . T . . . . . . . . . S - - .
PaTx-a    . . . . . . . . P . Q . . . G . . . . . . N I . . . Q E . L Q . K M N P . . . . . . . . . . . . . . . . . . . . . - - V . A . . A . T . . . . . . . . . S - - .
HrTx-a    . F . T A L E . G T F Q . . T . Y H . K . G I . A T Q D . T F T H . P S . Q G Y T G . S . . . . . A . D . . F . R - - . . I N N W V S . . D N D T F . L S E V .
NeoVTx-a  . F . T A L E . G . F Q . . S . Y H . K D G S . A T Q D . T F . R M P F . Q G Y K K . S . . . . . A . D . . F . R - - . . I N I W V S . . D N D T F . A S E H .
SNTX-a    . F I T A L E . G . F Q . . S . Y . . K . G S . A T Q D . T F . R M P F . Q G Y K K . S . . . . . A . D . . F . R - - . . I N N W I S . . D N D T F . A S E H .
IjTx-a    . F . . A L E . G . Y Q . . S . Y H . K . G S . A T Q D . T F . R M P F . Q G Y K N . S . . . . . A . D . . F N R - - . . I N I W V S . . D N D T F . A S E F A
SpCTx-b   . . . . . . . . P . E . . . . . . . . . . G I Q . I N D . S N . R . P P . . T . Q . Q E S - - - . S G M T V S S . W K E . A . . A . H . . . . . . . . M S - - .
SmTx-b    . . . . L . I . P . E . . . . . . . . . . G I Q . I N D . T K . Y . P P . K T . Q . . . E . . . . . . . . . . . . - - D . A . . A . T . . . . . . . . M S - - .
SoTx-b    . . . . . . . . P . E . . . . . . . . . . G I Q . I N D . S N . R . P P . . T . Q . . . E . . . . . . . . . . . . - - E . A . . A . H . . . . . . . . M S - - .
SsTx-b    . . . . . . I . P . E . . . . . . . . . . G I Q . I N D . S K . Y . P P . . T . Q . . . E . . . . . . . . . . . . - - E . A . . A . H . . . . . . . . . S - - .
PlTx-b    . . . . . . G . P . Q . . . G . . . . . . G I Q . I D E . T K . W . P P . . T . R . . . E . . . . . . . . . . . . - - D . A Q . A . T . . . . . . . . . S - - .
PvTx-b    . . . . . . G . P . Q . . . G . . . . . . G I Q . I D E . T K . W . P P . . T . Q . . . E . . . C . . . . . . . . - - D . A Q . A . T . . . . . . . . . S - - .
PaTx-b    . . . . . . G . P . Q . . . G . . . . . . G I Q . I D E . T K . W . P P . . T . Q . . . E . . . . . . . . . . . . - - D . A Q . A . T . . . . . . . . . S - - .
HrTx-b    . I . . . . Y D P . Q . . . G . . . . . . . I Q . I A E . T K . Q M P R . . A . K . . . E . F . . . . . . . . . . - - E . A . L A . A . . . . . . . . . S - - .
NeoVTx-b  . F . T A . N . P . Q . . . G . . . . . . . I Q . I H E . T K . H M P G . . T . K . . . E . Q . . . . . . . . . T - - E . A . Q V . T . . . . . . . . . S - - .
SNTX-b    . F . T A . H . P . Q . . . G . . . . . . . I Q . I D E . T K . Y M P G . . S . K . . . E . Q . . . . . . . . . . - - E . A . Q V . T . . . . . . . . . S - - .
IjTx-b    . F . T A . H . P . Q . . . G . . . . . . . I Q . I D E . T K . Y M P N . . T . K . . . E . Q . . . . . . . . . . - - E . A . K G . T . . . . . . . . . S - - .
                                                *

SpCTx-a   T L R A F D N N P E R F G L W Q Q V L C N K G L S R R H Y W E V E W N G Y V I V G V T Y S S I G R K N I D I Q S F I G F S E T S W T L M F I P K N G V A V K G A
SmTx-a    K Q Q K Y S V H . . . . N I F R E . . . K E . . N G Q . . . . . . . T . H . S A . . A . V G . A . . D F K P E . L . . . N L K . . A . C A T . . T . Y V I W - H
SoTx-a    K H Q K Y S K H . . . . D N Y R . . . . K E P . R G . . . . . L . . . . T . . A . . . . L . . . . . . . . . . . V . . . N . . . . . F I S F . . . . Y F I . . T
SsTx-a    K Q Q N Y S K H . . . . E N H R . . . . K E P . R . . . . . . . . . . . F . . . . . . . . . V . . . . L . . . . V . . . N . . . . . . . S F . R . . F F . . . .
PlTx-a    K P K P S . . . . . . . E H F . Y . . . K . Q . I . . . . . . I K . S . F . G A . . . R N . . H P . S S H V D . S L . G . D K . . V F E Y N . N T . Y R Q I - F
PvTx-a    K P K P S . . . . . . . E H F . Y . . . K . Q . I . . . . . . I K . S . F . G A . . I R N . . H P . S S Q V D . S L . G . D K . . V F E Y N . N T . Y R Q I - F
PaTx-a    K P K P S . . . . . . . E H F . Y . P . K . Q . I . . . . . . I K . S . F . G A . . R R N . . H P . S S Q V D . S L . G . D K . . V F E Y N . N T . Y R Q I - F
HrTx-a    K R Q N Y P K H . . . . L G Y N . . . . . E . . M G K . . . . . . . . . . . D . . I A . I . . P . . E A . F P . A . . Y N T Y . . I F R S Y . . . . Y L E L - H
NeoVTx-a  K R Q N Y P K H . . . . L C Y N . . . . . E . . T G K . . . . . . . . . . . D . . . A . I . . S . . E D N W V . A . . H N T C . . V F S S . . R A . Y V E R - Y
SNTX-a    K R Q N Y P K H . . . . V S F N . . . . . E . . M G K . . . . . . . . . . I D . . I A . I . . P . . E . . F A . A F . Y N T Y . . V . S Y N . . I . Y I E R - H
IjTx-a    K R Q N Y P K H . . . . I G Y N . . . . . E . . T G K . . . . . . . . . . . D . . . A . N . . D . . Q - . W V . A . . H N N C . . V V S S T . R I . Y . E R - Q
SpCTx-b   K P Q P S . . . . K . . D H Y . . . . . . . . . . K . . . . . . . . C . . . R A . I . . K G . Q . . T F A S E C S L . H T D M . . V F D Y Y . . S . Y H H I - Y
SmTx-b    E P K P . . . . . . . . K H H . . . . . D . . . . G . . . . . . . . S . . . C A . . . . R A . S . T E S . S G G L L . H N . G . . G F D Y Y . N S . Y N H I - S
SoTx-b    K P Q P S . . . . . . . D H Y . . . . . . E . . . K . . . . . . . . C . . . R . . I . . K A . Q . . T S . S V N A V . Q N L A . . A F D Y D . . S . Y C H I - Y
SsTx-b    K P Q P S . . . . . . . D H Y . . . . . . . . . . K . . . . . L . . C . . . R . . I . . K G M R . . T S . S V N A V . Q N L G . . A F D Y N . . S . Y S H I - Y
PlTx-b    K P K P . . . Q Q R . . E C Y . . . . . . . . . . . . . . . . . . . S . . . R A . . . . K G . S . R T W N Q D . S L . H N K I . . V F D Y F . . S . Y N H I - F
PvTx-b    . A K P . . . Q Q R . . Q C Y . . . . . . . . . . . . . . . . . . . S . . . R A . . . . K G . S . R T W N Q D . S L . H N K I . . V F D Y F . . S . Y N H I - F
PaTx-b    . A K P . . . Q Q R . . Q C Y . . . . . . . . . . . . . . . . . . . S . . . R A . . . . K G . S . R T W N Q D . S L . H N K I . . V F D Y F . . S . Y N H I - F
HrTx-b    K T K P Y . . H . . . . S H F . . . M . T . . . T G . . . . . L . . S C S . S A . . . . K G . . K N S S T S D . S L . Y N . K . . V . E Y S . . S . Y Q L I - E
NeoVTx-b  S T K S P A D H F . K . S H F . . . M . T . . . . G . . . . . L . . S . H . S A . . . . K G . S . . T S T P D . S L . K N Q K . . V F E Y T K . S . Y Q Q I - H
SNTX-b    N T K S P T D H L . K . S H F . . . M . T . . . . G . . . . . L . . S . . . G A . . . . K G . . . . T S T S D . S L . K N . K . . L F E Y S T . S . Y Q Q I - H
IjTx-b    N T K . S T D T I . N . S H F . . . M . I . . . . G . . . . . L . . S . D . G A . . . . K G . . . N T S N S D . S L . N N . K . . V F E Y S S . S . Y Q Q I - E
                                                                    \       \                                                           \

SpCTx-a   R R S V S Y Y F I S D L A F P P L G L Y H D C H A S T L S F Y K V - S D N V L N H F H T I E I K P K L S E P V Y A I I R I G D E D R P Y H G T V R L L - -
SmTx-a    N Q E K V L V S M P . P N . T R . . V . L . W R . G S . . . . M . - . S . . . H . L W . - - F . T . F T . . . . P A F C . . L M Q - - F N . K A . . . - -
SoTx-a    G I E L L R V G L P . P D . T R . . V . L . . . . . . . . . . . . - . S . . . H . . . . . . . . . . . . . . . . . . . . . . V . . . . . . R A . . . - -
SsTx-a    D M . P V R I G L P . P D . T R . . V F L . H . . . . . . . . . . - . S . . . H . . . . . . . . . Q . . . . . . . . . . . . . V . V . L . . S . . . . - -
PlTx-a    K N . P E N V N V . S T G . D R Q . V . L . W L G G . . . Y . M I - N N . F V T . L . . - - F H T L F K . A . . P A F M . . . K Q - N V . . E I E . . - -
PvTx-a    K N T P E N V N V . S T G . D R . . V . L . W L G G . . . Y . M I - N N . F V T . L . . - - F H T L F K . A . . P A F M . . . K Q - N V . . E I E . . - -
PaTx-a    K N T P E N V N V . S T G . D R . . V . L . W L G G . . . Y . M I - N N . F V T . L . . - - F H T L F K . A . . P A F M . . . K Q - N V . . E I E . . - -
HrTx-a    N K K E T H V R V T T P G . K R . . V F L . W R G G S . . . . A . - . S . E V R . L . . - - . . T . F T . . . . P A F G . . I G . - - . . . S L . . V - -
NeoVTx-a  N Q R Q Y . V T V P T P G . K Q . . V F L N W P D G S . . . . A . - . S D E V H . L . . - - F . T . F T . . . . P A F C L . Y R F - - D . . . . . . . - -
SNTX-a    K K R E Y N V R A P N P G . K R . . . F L . W R Y G S I . . . A . - . S D E V H . L . . - - F . T . F T . . . . P A F S . . P A G - - N . . . L . . . - -
IjTx-a    Q . K Q N . F R V P N P V . K Q . . V F L N W C D G S . . . . S . S . S D E V H . L . . - - F . T . F T . . . . P A F C . . Y R L - - D N . S . . . . - -
SpCTx-b   N N K K V R V K V A S P G . D R . . V . L . W P . G . . . . . M . - T S T W V T . L . . - - F S I R F N . A . . P A F L . . H G Q K N A N . Q I K . K G E
SmTx-b    N N Q K L T V T V . S P G . A R . . V . L . W P . G . . . Y . M . - T T T W V T . L . . - - F Q T R F T . A . . P G F Q F . . P K L N C K . Q I K . . - -
SoTx-b    G N E K T P V K V A S P G . D R . . V . L . W P . G . . . . . M . - T S T W V T . L . . - - F S A . F Y . A . . P A F L . . . A Q Q K T N . Q I K . . - -
SsTx-b    G N E K . S V K V A S P G . D Q . . V . L . W P . G . . . . . M . - T S T W V T . L . . - - F S A . F I . A . . P A . L . . . G Q Q K T N . Q I K . . - -
PlTx-b    D A K K T K V N V . S P G . K K . . I . L . W P . G . . . . . M . - D T I W V T . I . . - - F . A R F Q . A . F P A F L . . H G S - - D N . Q I K . . - -
PvTx-b    D A K K T K V N V . S P G . K K . . I . L . W P . G . . . . . M . - D T I W V T . I . . - - F . A R F Q . A . F P A F L . . H G P - - D N . Q I K . . - -
PaTx-b    D A K K T K V N V . S P G . K K . . I . L . W P . G . . . . . M . - D T I W V T . I . . - - F . A R F Q . A . F P A F L . . H G P - - D N . Q I K . . - -
HrTx-b    N G K K T R V S V . S P G . E Q . . V . L . W P . G . . . . . M . - . K A W V T . L . . - - . . T . F Y . A . . P A F L . . N A Q - - Q N . R I K . . - -
NeoVTx-b  N G K N A R V T V . S I G . K Q . . V . L . W P . G . . . . . M . - N K A W V T . L . . - - F H T . F Y . A . . P A F L . . . A Q Q K V N . Q I K . . - -
SNTX-b    N S K K T R V T V . S T G . K L . . V . L . W P . G . . . . . M . - N K A W V T . L . . - - F H T . F N . A . . P A F L . . . A Q Q K V N . Q I K . . - -
IjTx-b    N G K K T R V T V . S T G . K Q . . V . L . W P . G . . . . . M . - K K P W V T . L . . - - F R T . I H . A . F P A F H . . . A Q K K T N . Q I K . . - -
                                                                                          *
```
